# Adverse childhood experiences in the children of the Avon Longitudinal Study of Parents and Children (ALSPAC)

**DOI:** 10.12688/wellcomeopenres.14716.1

**Published:** 2018-08-30

**Authors:** Lotte C. Houtepen, Jon Heron, Matthew J. Suderman, Kate Tilling, Laura D. Howe

**Affiliations:** 1 MRC Integrative Epidemiology Unit, University of Bristol, Bristol, BS8 2BN, UK

**Keywords:** childhood adversity, trauma, ALSPAC, ACE, maltreatment

## Abstract

**Background: **Exposure to adverse childhood experiences (ACEs) is a risk factor for poor later life health. Here, we describe the ACE variables measured in the children of the Avon Longitudinal Study of Parents and Children (ALSPAC) study, and a method used to derive summary measures and deal with missing data in them.

**Methods: **The ALSPAC data catalogue (59 608 variables) was searched in September 2017 for measures on adversity exposure between birth and 18 years. 6140 adversity questions were then screened for conforming to our ACE definitions and suitability for dichotomisation. This screening identified 541 questions on ten ‘classic’ ACEs (sexual, physical or emotional abuse, emotional neglect, substance abuse by the parents, parental mental illness or suicide attempt, violence between parents, parental separation, bullying and parental criminal conviction) and nine additional ACEs (bond between parent and child, satisfaction with neighbourhood, social support for the parent, social support for the child, physical illness of a parent, physical illness of the child, financial difficulties, low social class and violence between child and partner). These were used to derive a binary construct for exposure to each ACE. Finally, as cumulative measures of childhood adversity, different combinations of the 19 ACE constructs were summed to give total adversity scores. An appropriate strategy for multiple imputation was developed to deal with the complex patterns of missing data.

**Results: **The ACE constructs and ACE-scores for exposure between birth and 16 years had prevalence estimates that were comparable to previous reports (for instance 4% sexual abuse, 18% physical abuse, 25% bullied, 32% parental separation).

**Conclusions: **ACE constructs, derived using a pragmatic approach to handle the high dimensional ALSPAC data, can be used in future analyses on childhood adversity in ALSPAC children.

## Introduction

Exposure to Adverse Childhood Experiences (ACE) is associated with substantial health consequences ^1, 2^. Studies show a graded relationship between ACEs and poor outcomes, with the more ACEs a person suffers the greater their risk for many health conditions (for meta-analysis see 2). The most commonly examined ACEs ^3^ include child maltreatment (e.g. emotional, physical and sexual abuse as well as physical or emotional neglect) and broader experiences of household dysfunction (e.g. violence between parents, parental separation and household affected by substance misuse, mental illness or criminal behaviour), although it has been argued other types of adversities (e.g. bullying, poverty, neighbourhood violence) should be included ^4, 5^ and many ACE studies incorporate additional adversities ^2^.

Our goal was to derive measures for childhood adversity for the children of a British birth cohort study, the Avon Longitudinal Study of Parents and Children (ALSPAC). In this cohort, a vast array of detailed adversity data has been obtained from multiple parent- and child-completed questionnaires administered throughout childhood and adolescence. Using these data presents challenges given the repeated measures, differences in measurement tools across time, and complex missing data patterns. Given the known co-occurrence of multiple forms of adversity and the potential presence of cumulative effects on health ^2^, we also derive ACE count score measures. In this Data Note, we describe the processes used to derive the ACE measures and resources available for researchers to use in their own studies, and we provide descriptive statistics of the ACE measures.

## Methods

### ALSPAC sample

ALSPAC recruited 14,541 pregnant women resident in Avon, UK (former county covering Bristol and the surrounding areas in the South West UK) with expected dates of delivery 1st April 1991 to 31st December 1992. Each enrolled mother either returned at least one questionnaire or attended a “Children in Focus” clinic by 19/07/99. Of these initial pregnancies, there were a total of 14,676 foetuses, resulting in 14,062 live births and 13,988 children who were alive at 1 year of age.

When the oldest children were approximately 7 years of age, an attempt was made to bolster the initial sample with eligible cases who had failed to join the study originally. As a result, when considering variables collected from the age of seven onwards there are data available for more than the 14,541 pregnancies mentioned above ^6, 7^.

The total sample, including later enrolment phases, is 14,775 live births and 14,701 alive at 1 year of age. Note that for reasons of confidentiality questionnaire data belonging to children from triplet or quadruplet pregnancies have been removed, resulting in 14,691 eligible participants.

The mothers, their partners and the index child have been followed-up using clinics, questionnaires and links to routine data. Please note that the study website contains details of all the data that is available through a fully searchable data dictionary.

We restricted our derivation of ACE measures to the 12087 children who answered at least 10% of the 541 questions on ACE exposure between 0–18 years.

Ethical approval for the study was obtained from the ALSPAC Law and Ethics Committee and the Local Research Ethics Committees.

### ACE questions

Based on previous literature on ACEs ^2– 5^, previous research on childhood adversity in ALSPAC ^8– 12^ and discussion between LDH and LCH on ACEs to include, we searched for ALSPAC data on 20 ACEs. Ten of the twenty ACEs are frequently used in other research ^3^: i. sexual abuse, ii. physical abuse, iii. emotional abuse, iv. emotional neglect, v. substance abuse by the parents, vi. parents have mental illness or attempted to commit suicide, vii. violence between parents, viii. parental separation, ix. bullying and x. parent convicted of an offence, whereas the other nine ACEs were either suggested more recently ^5^, examined in ALSPAC before ^9^ or identified as relevant by LDH and LCH when deciding which ACEs to include: xi. bond between parent and child, xii. satisfaction with neighbourhood, xiii. social support for the parent, xiv. social support for the child, xv. physical illness of a parent, xvi. physical illness of the child, xvii. financial difficulties, xviii. low social class, xix. violence between child and partner and xx. crowded housing.

In September 2017, text searches (see Supplementary File 1 for keywords used) as well as a visual scan of the ALSPAC data dictionary (59608 variables) were performed by LCH to identify variables of interest. The resulting 12083 questions were compared to previous articles using ALSPAC data to ensure that we had identified all relevant variables ^8– 12^. 6487 of the 12083 questions were classified into the 20 ACEs. After careful examination of the questions and response possibilities by LCH and LDH, questions were excluded if they did not conform to our ACE definitions (see section ‘ACE definitions’) or were unsuitable for dichotomisation. Crowded housing was the only ACE that was not included due to the limited number of questions on crowding and coverage of a small age range (birth- 3 years).

Of the 644 remaining questions (see Supplementary Table 1; Supplementary File 1), the 541 questions that covered exposure to nineteen ACEs between 0–18 years can be used for ACE derivation (overview of the types of variables in Table 1), whereas the other variables on ACE exposure before birth or after 18 years are recommended auxiliary variables for multiple imputation (see paragraph on ‘Multiple imputation’). 41 of these 541 ALSPAC questions ask about ACE exposure in two different time periods (e.g. the answer options for one question were ‘occurred when the child was 6 or 7 years’, ‘since 8th birthday’, ‘both time periods’ or ‘did not happen in past 3 years’). To enable examination of exposure in different time periods, each of these questions was split into two dummy variables (e.g. exposure at age 6 or 7 and exposure at age 8 years), resulting in 582 ACE variables for exposure between 0–18 years. The majority of the early life data (0–8 years) is parent reported, but when the children were 8 years old they began self-reporting ACEs. Moreover, in their twenties, the participants retrospectively reported on child maltreatment (several forms of abuse and neglect), violent behaviour of their own partner as well as whether their parents were violent towards each other. Overall 89% of all ACE variables were collected prospectively, but we also included retrospective self-report measures as these complement the prospective data. For instance, the sexual abuse rates prospectively reported by parents were much lower than those retrospectively self-reported by the participants.

**Table 1.** The number of variables used to derive each adverse childhood experience (ACE), and the percentage of these that are based on prospective (versus retrospective) and child (versus parent) reported data.

	*Number* *of* *variables*	*Prospective* *variables* *(n (%))*	*Child reported* *variables* *(n (%))*
**CLASSIC ADVERSE CHILDHOOD EXPERIENCES**			
**physical abuse**	49	32 (65%)	9 (18%)
**sexual abuse**	12	7 (58%)	5 (42%)
**emotional abuse**	46	33 (72%)	5 (11%)
**emotional neglect**	23	20 (87%)	20 (87%)
**bullying**	19	19 (100%)	19 (100%)
**substance household**	70	70 (100%)	1 (1%)
**violence between parents**	48	44 (92%)	0 (0%)
**parental mental health** **problems or suicide**	82	78 (95%)	2 (2%)
**parent convicted offence**	25	21 (84%)	0 (0%)
**parental separation**	48	39 (81%)	3 (6%)
**EXTENDED ADVERSE CHILDHOOD EXPERIENCES**			
**social class**	6	6 (100%)	0 (0%)
**financial difficulties**	44	40 (91%)	0 (0%)
**satisfaction with** **neighbourhood**	11	11 (100%)	5 (45%)
**social support of parent**	14	14 (100%)	0 (0%)
**social support of child**	17	17 (100%)	17 (100%)
**violence between child and** **partner**	13	6 (46%)	13 (100%)
**physical illness of the child**	11	11 (100%)	0 (0%)
**physical illness of a parent**	23	23 (100%)	0 (0%)
**parent-child bond**	21	20 (95%)	3 (14%)

### ACE definitions

The exact phrasing, definition and time of collection for variables on all nineteen ACEs is described in Supplementary Table 2 ( Supplementary File 1), but in short:

1. ever sexually abused, forced to perform sexual acts or touch someone in a sexual way ( *sexual abuse*);
2. adult in family was ever physically cruel towards or hurt the child ( *physical abuse*);
3. parent was ever emotionally cruel towards the child or often said hurtful/insulting things to the child ( *emotional abuse*);
4. child always felt excluded, misunderstood or never important to family, parents never asked or never listened when child talked about their free time ( *emotional neglect*);
5. parent was a daily cannabis or any hard drug user, or, had an alcohol problem ( *substance use*);
6. parent was ever diagnosed with schizophrenia or hospitalised for a psychiatric problem, or, during the first 18 years of the child’s life, parent had an eating disorder (bulimia or anorexia), used medication for depression or anxiety, attempted suicide or scored above previously established cut-offs for depression (Edinburgh Postnatal Depression Scale (EPDS) >12 ^13^) ( *mental health problems or suicide*);
7. parents were ever affected by physically cruel behaviour by partner, or, ever violent towards each other, including hitting, choking, strangling, beating, shoving ( *parents violent towards each other*);
8. parents separated or divorced ( *parental separation*);
9. child was a victim of bullying on a weekly basis ( *bullying*);
10. parent was convicted of a crime ( *parent convicted*);
11. child or parent not close to each other and when growing up, child never felt loved ( *parent-child bond*)
12. child is not happy living in neighbourhood or would rather move, parent or child describe neighbourhood as bad ( *satisfaction with neighbourhood*);
13. parent never had anyone to share feelings with ( *social support of parent*);
14. child has no friends, unhappy with number of friends or friends hardly ever support them ( *social support of child*);
15. parent hospitalised more than once or had cancer ( *physical illness of a parent*);
16. child hospitalised more than once or had a medical condition or physical disability ( *physical illness of the child*);
17. very difficult to afford food or heating, or, parent was affected by becoming homeless ( *financial difficulties*);
18. highest household social class was in class V (unskilled work) or unemployed, based on mother’s and her partner’s occupations using the 1991 UK Office of Population Censuses and Surveys classification (classes I to V) ( *social class*);
19. partner of child used physical force or violence, or, made them feel scared ( *violence between child and partner*);

### Derive ACE measures

The 582 ACE variables were recoded to a binary yes/no based on pre-set criteria ( Supplementary Table 1; Supplementary File 1). Only four of the 12083 participants had data for all 582 ACE variables. This is unsurprising considering the sensitive nature of the ACE questions as well as the diverse variables included (collected at many different time points over a long period (birth-23 years) from various informants (mother, child, mother’s partner) with different methods (clinics, questionnaires)). Ideally, we would use multiple imputation to impute missing values of individual ACE-related questions, but the lack of complete cases in combination with the high number of variables would lead to a multi-dimensional imputation model that could not converge. Therefore, we opted for a pragmatic approach. We derived a binary construct for exposure to an ACE during a certain time period if the participant answered more than 50% of the questions for that ACE in the specified time period (e.g. answered at least six of the twelve questions on exposure to sexual abuse between 0–16 years). For participants who had responded to <50% of the questions, we coded the binary ACE construct as missing. From here on we refer to the participants that had enough data to derive the ACE as ‘ACE-derived’. Further details of the imputation procedure are provided below.

Similar to previous studies ^1, 2^, a cumulative adversity measure was derived by summing exposure to the ten classic ACEs (ACE-score) and dividing the ACE-score into four categories (0, 1, 2-3 and more than 4 ACEs). Similarly, an extended ACE-score (summing exposure to all nineteen ACEs) and a categorical extended ACE-score (0-1, 2, 3-6 and more than 6 ACEs), with a similar distribution to the original scores, were derived.

The R code used to derive these ACE measures in R 3.3.1 will be supplied together with the data. We derived ACE measures for the time period 0–16 years, as this is a frequently used time period in previous ACE studies ^2^, but the code can be readily adapted to different time periods between 0–18 years depending on a researcher’s needs. However, note that questions that span a larger time window (e.g. exposure 0–11 years or 0–16 years) than the period of interest (e.g. 0–8 years) would be excluded from ACE construct calculations. The questions and cut offs used to derive the dataset described below are supplied in Supplementary Table 1 and Supplementary Table 2 ( Supplementary File 1).

### Multiple imputation

ACE measures were derived for participants who responded to at least 50% of the questions (ACE-derived); these participants are more affluent than the full cohort, and including only these participants in analyses will lead to lower ACE prevalence estimates and may induce selection bias ^14^. Therefore, for multiple imputation we recommend including two types of auxiliary variables that make the missing-at-random assumption more plausible (see Supplementary Table 3):

1. sociodemographic indicators that are associated with both missingness and many of the ACEs ( Supplementary Table 6 and Supplementary Table 7; Supplementary File 1) (ethnicity of the child, maternal age at birth, mother’s home ownership status at birth, parity, maternal marital status at birth, mother and partner’s highest educational qualification, maternal EPDS score at 18 and 32 weeks gestation and mother’s partner’s EPDS score at 18 weeks gestation, birthweight, gestational age, maternal weight, maternal BMI, maternal smoking during pregnancy)
2. adversity questions on ACE exposure either before birth (mother became homeless, mother taking medication for depression, parents’ EPDS score, mother’s opinion of neighbourhood, partner of mother was convicted of offence, mother separated from partner, partner’s hard drug use, mother had difficulty affording heat or food, highest household social class) or between 18–21 years (mother’s partner was emotionally cruel to child, maternal antidepressant use, mother separated from partner, partner of child was violent towards child, parent’s alcohol use disorders identification score (AUDIT)). However, as adversity exposure may be rare we recommend only including questions with at least 50 adversity exposed participants in your own imputation model.

To illustrate the use of multiple imputation using these two types of auxiliary variables, we compare prevalence estimates for the imputed ACE constructs for exposure between 0–16 years to the prevalence estimates in the ACE-derived group. To preserve potential interactions between gender and adversity in relation to later outcomes, males (n=6214) and females (n=5873) were imputed separately before appending the two datasets. For both males and females, 90 imputed datasets were created using the mice package version 2.46.0 in R3.3.1 with 30 iterations per dataset ^15^, based on the rule of thumb that the number of imputed datasets should be at least equal to the percentage of incomplete cases (9% for all 19 ACEs, 29% for ten classic ACEs) and for some variables the imputation model converged after 20 iterations ^16^.

Do note that the multiple imputation process will need to be re-done for any future applications because the imputation model would have to be compatible with the analysis model(s) being used, so to avoid bias the imputation model should include the exposure, outcome and any covariates, plus any interactions and non-linearities ^16^. For instance, our multiple imputation was carried out separately for males and females to enable examination of gender interactions in the imputed data, but to examine other interactions the imputation model would have to be adapted to reflect this.

### Patterns of missing data and prevalence of ACEs

Consistent with a higher missingness rate in more deprived participants ^14^, sociodemographic indicators (parental education, social class and home ownership) were lower in the ACE-derived group ( Supplementary Table 4; Supplementary File 1). As expected, ACE prevalence estimates are higher in the imputed data ( Table 2). In line with socially patterned adversity exposure, the imputed ACE prevalence estimates were higher in children from a lower social class ( Supplementary Table 6; Supplementary File 1) and lower in children of highly educated women ( Supplementary Table 7; Supplementary File 1).

**Table 2.** Prevalence estimates for the ACE measures in the ACE-derived group as well as the imputed sample.

Variable	ACE-derived group (participants with data for at least 50% of ACE questions between 0–16 years)		Imputed data (N=12087)
	N	Mean (SE) for continuous variables % for categorical variables	Mean (SE) for continuous variables % for categorical variables
**ACE-score**	3598	1.77 (0.03)	2.18 (0.02)
**Categorical ACE-score** *0* *1* *2–3* *4+*	3598	22.3 28.7 35.1 13.9	17.4 25.2 36.1 21.4
**physical abuse**	6447	14.9	17.6
**sexual abuse**	9120	2.8	3.7
**emotional abuse**	6921	19.3	22.5
**emotional neglect**	5716	19.3	22.1
**bullying**	7071	24.2	25.3
**parents violent towards** **each other**	6419	19.1	24.1
**substance household**	7371	9.4	13.7
**parental mental health** **problems**	7381	42.7	47.0
**parent convicted**	7656	7	9.4
**parental separation**	6603	25.3	32.2
**Extended ACE-score**	1109	3.00 (0.07)	3.58 (0.02)
**Extended categorical** **ACE-score** *0–1* *2* *3–5* *6+*	1109	25.4 23.2 39 12.4	29.1 22.5 47.1 1.4
**social class**	5605	9.6	11.7
**financial difficulties**	7629	13.6	18.4
**satisfaction with** **neighbourhood**	8805	8.9	10.8
**social support parent**	6703	10.8	12.6
**social support child**	7935	11.1	13.8
**violence between child** **and partner**	4003	10.8	13.9
**physical illness of the** **child**	9292	8.9	9.9
**physical illness of a** **parent**	5875	24.1	27.1
**parent-child bond**	6842	19.4	22.5

In the imputed data, the classic ACE-score categories 0, 1, 2–3 and 4+ represented respectively 17, 25, 36 and 21% of the population ( Table 2). Sexual abuse had the lowest prevalence (3.7%), whereas having a parent with mental health problems was most common (47%). Most of our ACE estimates were within a similar range to prevalence estimates reported in the New-Zealand based Dunedin birth cohort study ^17^ or previous UK based ACE studies ^2, 18– 20^. Only parental mental health problems (41%), also our most prevalent ACE, was much higher than other ACE studies but still in line with lifetime mental health prevalence estimates in the US (Kessler *et al*., 2005) and Northern Ireland (Bunting, Murphy, O ’neill, & Ferry, 2012). The correlation (Cramér's V for nominal variables) between the individual ACEs varied from low to medium with ϕc ranging 0 to 0.32 ( Supplementary Figure 1; Supplementary File 1). Overall, emotional abuse, parental separation and violence between parents had the strongest correlations with other ACEs (ϕc>0.2 for at least four other ACEs). The strongest correlation was present for physical abuse and emotional abuse (ϕc=0.32).

Females were more likely to experience physical abuse and sexual abuse than males (respectively 19% and 5% in females versus 16% and 2% in males), and were less likely to experience emotional neglect, bullying, lack of social support, violence between child and partner and physical illnesses (respectively 20%, 23%, 10%, 12% and 7.5% in females versus 24%, 28%, 15%,16%,12% in males) ( Supplementary Table 5; Supplementary File 1).

## Dataset validation

**Figure 1.**
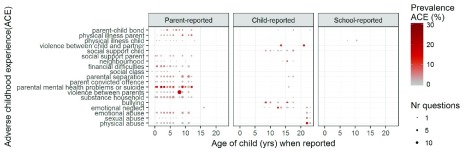
Prevalence of each ACE by age when reported, data source (parent, child or school) and number of individual questionnaire items. Each ACE prevalence was calculated for the participants that answered at least 50% of the questions for the ACE measure at each age of reporting and data source.

Detailed, repeated measures of childhood adversity are available in the ALSPAC study. We extracted 582 variables documenting exposure to 19 different ACEs between birth and 18 years. Overall, ACE exposure prevalence did not differ by age of reporting or data source (indicated by similarly coloured circles in Figure 1). The higher prevalence for violence between parents at age 8 compared with other ages is likely related to the inclusion of more detailed questions at this time point.

Our definitions for the ten classic ACEs were very similar to the most commonly used definitions ^3^, although, due to the data available, we focused on parents being convicted of a criminal offense, instead of incarceration of a parent. However, in this population-based setting the rate for convicted parents was less than 10%, thus it is unlikely incarceration would have been sufficiently prevalent to be examined as a separate ACE. In addition to the ten classic ACEs, we also derived several other types of ACEs. The reason for this is a growing body of literature showing there are other types of adversities that cluster with the original ACEs with a similar cumulative influence on health ^5^.

Most of our ACE estimates are comparable to other studies ^17, 21^, although in some previous UK population-based studies prevalence estimates tended to be slightly lower ^2, 18– 20^. Our higher rates could be due to the large number of data collection time points and mix of prospectively collected and retrospectively reported data from different reporters.

However, by using data from different sources over a large time period, missingness was a problem that had to be addressed. Importantly, it was not possible to even use a complete case approach. Furthermore, owing to the complex missing data pattern and large number of adversity variables, we had to take a pragmatic approach to imputation. We first calculated the ACE constructs for participants with at least 50% of the questionnaire items for each particular ACE (ACE-derived group). The remainder were coded as missing and imputed using multiple imputation. This assumes that the data are missing-at-random given the variables included in the imputation model ^16^. Although this assumption is untestable, it allows for maximum use of the available ACE data, and we included a number of key sociodemographic variables in the imputation model to make this assumption more plausible. Any bias in the imputation model is likely to lead to underestimation of the ACE prevalence and potentially biases analyses towards the null ^14^. 

In our implementation of the ACE framework, we dichotomised all ACE variables based on pre-defined cut offs to capture exposure and derived an ACE count score measure that is widely used in literature as a summary variable. A limitation to our implementation is that the summing procedure implicitly assumes that each ACE has the same direction and magnitude of effect on outcomes ^22^. Also, there is more detail in the ALSPAC data that could be exploited, for instance on the subjective impact of ACE exposure. Researchers wishing to use these more detailed data could do so, but this would necessitate further data manipulation. Finally, we relied on the questionnaire and clinical childhood adversity data, but ALSPAC also has linkage data available on looked after children ^9^ and there is future potential of linkage to criminal convictions and cautions.

Although other software packages can be used to derive the ACE measures, together with the data we will provide the R code we used to (1) dichotomise the variables, (2) derive the ACE measures for a specific time period and (3) implement multiple imputation.

Overall, we describe a pragmatic method for deriving ACE constructs using a wealth of data on a UK population-based sample. Missing data was a key issue that needed to be handled, which is why, for future analyses with these ACE measures, we advise using multiple imputation by adapting the framework detailed in this Data Note.

## Ethics policies

Ethical approval for the study was obtained from the ALSPAC Ethics and Law Committee and the Local Research Ethics Committees. A comprehensive list of research ethics committee approval references is available to download at: http://www.bristol.ac.uk/alspac/researchers/research-ethics/.

## Data availability

ALSPAC data access is through a system of managed open access. The steps below highlight how to apply for access to ALSPAC data, including access to the data and R scripts described in this data note .

1. Please read the ALSPAC access policy (PDF, 627kB) which describes the process of accessing the data and samples in detail, and outlines the costs associated with doing so.

2. You may also find it useful to browse our fully searchable research proposals database, which lists all research projects that have been approved since April 2011.

3. Please submit your research proposal for consideration by the ALSPAC Executive Committee. You will receive a response within 10 working days to advise you whether your proposal has been approved.

If you have any questions about accessing data, please email alspac-data@bristol.ac.uk.

The ALSPAC data management plan describes in detail the policy regarding data sharing, which is through a system of managed open access.

## Consent

Written informed consent was obtained from the parents of participating children after receiving a full explanation of the study. Children were invited to give assent where appropriate. Study members have the right to withdraw their consent for elements of the study or from the study entirely at any time. Full details of the ALSPAC consent procedures are available of the study website.

## Supplementary material

Supplementary File 1: Word document containing supporting text (on the data search, household social class derivation and auxiliary imputation variables), seven Supplementary tables (on ACE definitions, imputation variables, gender differences and ACE variables) and a Supplementary figure (on the correlation between different ACE constructs).
