# Supplementary material for: Adverse childhood experiences in the children of the Avon Longitudinal Study of Parents and Children (ALSPAC)
Source: Wellcome Open Res. 2018 Aug 30;3:106. [Version 1] doi: 10.12688/wellcomeopenres.14716.1 (PMC6281007; doi:10.12688/wellcomeopenres.14716.1)
Supplement: Supplementary file 1 [file wellcomeopenres-3-16031-s0000.tgz › 16d40fbb-4940-459f-8506-f82fdba5853a_supplementary_file_1.docx]

**Supplementary material for:**

**Adverse childhood experiences in the children of the Avon Longitudinal Study of Parents and Children (ALSPAC)**

L.C. Houtepen^1^, J. Heron^1^, M.J. Suderman^1^, K. Tilling^1^, L.D. Howe^1#^

^1^ MRC Integrative Epidemiology Unit at the University of Bristol, Population Health Sciences, Bristol Medical School, University of Bristol, Bristol, UK.

^#^Corresponding author: laura.howe@bristol.ac.uk. MRC Integrative Epidemiology Unit, University of Bristol, Oakfield House, Bristol BS8 2BN, UK. +44 (0)117 3310134

[Data search 2](#_Toc522134974)

[Household social class 2](#_Toc522134975)

[ACE definitions 3](#_Toc522134976)

[Imputation model 6](#_Toc522134977)

[Prevalence estimates for variables in imputation model for ACE_0-16yrs_ 10](#_Toc522134978)

[Gender differences ACE_0-16yrs_ 15](#_Toc522134979)

[Social class differences ACE_0-16yrs_ 17](#_Toc522134980)

[Maternal education differences ACE_0-16yrs_ 19](#_Toc522134981)

[Correlation between ACE constructs 21](#_Toc522134982)

[ACE questions 22](#_Toc522134983)

### Data search

The text word search was done using the grep function in R to search for the following regular expression in the variable description:

‘sex|emot|sworn|shake|help|financi|confid|cruel|into.*care|dying|living|burgl|vandal|domin|telling|calmly talk| order.*par|control|change.*par| med|binge|pint|touch|support|share|cuddle|home|trouble| household|qualification|employ|beer|spirit|wine|drinks|overdos|ecstasy|methad|crack|substance|barbitura|upset|underst|joy|unhappy|poor|abuse| event |phys|health|diab|strok|hypertension|heart disease| died|ill|death|viol|hit|slap|knife|choke|beate|throw|twist|growing up|close.*to|interaction|convers.*time|hurtful|insult|shout|bull|threat| lie|didn.t.*want|did.*not.*want|ask.*school|ask.*time|feel.*left out|underst.*by|feel.*imp|talk.* to|talk.* about|discuss.*probl|diff.*pay|feel scared|intentional|pressure|afford|homeless|medical condition|neighbourhood| live |hurt themselves|suicide|depres|epds|manic|schizo|illness|paranoid|anxi|anorexia|bulimia|same partn|convict|friends|social class|cancer|hosp|smack|shov|separ|divor|went away|is the same as|drink problem|alcohol|ganj|Canna|Cocai|Amphet|heroi|drug|audit’

### Household social class

Sixteen mother and partner reported measures of social class based on the 1991 UK Office of Population Censuses and Surveys classification (classes I to V, with III split into manual and non-manual) were combined to derive highest household social class at eight time points (12-weeks’ gestation, 32-weeks’ gestation (used as a proxy for social class at birth), eight months, two years, three years, four years and eight years). If multiple measures were available at the same time point, the highest reported measure was taken even if one of the measures was missing.

### ACE definitions

*Supplementary table 2*

| **Adverse childhood experience (ACE)** | **Phrasing** | **Criterium dichotomisation** | **Retrospective** |
| --- | --- | --- | --- |
| **Sexual abuse** | Sexually abused | yes | no |
|  | When growing up someone molested respondent (sexually) | yes | yes (asked at 23yrs) |
|  | Touched in a sexual way by adult or older child, or was forced to touch adult or older child in a sexual way, before age of 11 | yes | yes (asked at 22yrs) |
|  | Touched in a sexual way by adult or older child, or was forced to touch adult or older child in a sexual way, between ages of 11 and 17 | yes | yes (asked at 22yrs) |
|  | Adult or older child forced, or attempted to force, respondent into any sexual activity by threatening or holding respondent down or hurting respondent in some way, before age of 11 | yes | yes (asked at 22yrs) |
|  | Adult or older child forced, or attempted to force, respondent into any sexual activity by threatening or holding respondent down or hurting respondent in some way, between ages of 11 and 17 | yes | yes (asked at 22yrs) |
| **Physical abuse** | Partner/respondent was physically cruel to child | yes | no |
|  | Adult in family pushed, grabbed, shoved/smacked to discipline respondent, before age of 11 | often | yes (asked at 22yrs) |
|  | Adult in family pushed, grabbed, shoved/ smacked to discipline respondent, between ages 11 and 17 | often | yes (asked at 22yrs) |
|  | When growing up people in respondent's family hit them so hard that it left them with bruises or marks | yes | yes (asked at 23yrs) |
|  | Adult in family kicked, punched, hit respondent (so hard it left bruises or marks), before age of 11 | yes | yes (asked at 22yrs) |
|  | Adult in family kicked, punched, hit respondent (so hard it left bruises or marks), between ages 11 and 17 | yes | yes (asked at 22yrs) |
| **Emotional neglect** | Carer knows who friends are | never | no |
|  | Carer asks/starts conversation about free time/ what happened at school | never | no |
|  | Carer takes time to listen when teenager talks about what happened in free time | never | no |
|  | Discuss problems with anyone in their family | very difficult | no |
|  | Parent/carer talked about child's experiences at school/ friends/ things that are troubling | never | no |
|  | Child feels left out of things | always | no |
|  | Understood by parents | not | no |
|  | When growing up there was someone to take respondent to the doctor if needed | never | yes (asked at 23yrs) |
|  | Someone in family made child feel important or special, before 11 | never | yes (asked at 22yrs) |
|  | Someone in family made child feel important or special, between ages 11 and 17 | never | yes (asked at 22yrs) |
|  | Carer knows what child does with other children | nothing | no |
| **Emotional abuse** | Partner/respondent was emotionally cruel to child | yes | no |
|  | Adult in family shouted/ said hurtful or insulting things to respondent, before age of 11 | Very often | yes (asked at 22yrs) |
|  | Adult in family shouted/ said hurtful or insulting things to respondent, between the ages of 11 and 17 | Very often | yes (asked at 22yrs) |
| **Parental separation** | Parent reports divorce/separation | yes | no |
|  | Your parents have divorced/separated | yes | no |
|  | Parent still has the same partner/husband | no | no |
| **Mental health and suicide (attempts) by parents** | Parent has hurt themselves on purpose | yes | no |
|  | Parent has attempted suicide | yes | yes, the reported age can be retrospective |
|  | Taken medication for anxiety or depression | yes | no |
|  | Edinburgh Postnatal Depression Scale (EPDS) | >12 | no |
|  | Schizophrenia | yes, either current or ever | Both |
|  | Bulimia, anorexia nervosa | yes, recently | Both |
|  | Ever admitted to hospital for psychiatric or mental health problems | yes | yes |
| **Parents violent towards each other** | Physically cruel | yes, affected them | yes, the reported age can be retrospective |
|  | Aware of and affected by one 'parent' slapping, kicking, hitting or otherwise physically hurting the other | yes, ever | yes (asked at 21yrs) |
|  | Kicked, bitten or hit each other | yes | no |
|  | Physically twisted arm | yes | no |
|  | Throw(n) bodily | yes | no |
|  | Beaten each other up | yes | no |
|  | Choke or strangle each other | yes | no |
|  | threatened each other with knife | yes | no |
|  | Used knife or other weapon on each other | yes | no |
| **Substance abuse in household** | Smoked cannabis | every day | no |
|  | Hard drug use (including crack, heroin, amphetamine, opiate, cocaine, methadone, meth) | yes | no |
|  | Hard drug addiction | yes, recently | no |
|  | Alcoholism/ Drink problem | yes, ever / yes, saw doctor | no |
|  | Alcohol Use Disorders Identification Test (AUDIT) score | >8 | no |
| **Parent convicted** | Court conviction | yes | no |
|  | Convicted of an offence | yes | yes, the reported age can be retrospective |
| **Socioeconomic status (SES)** | Social class based highest occupation mother or father | Unskilled work | no |
| **Physical illness parent** | Hospitalised | >1 hospitalisation | no |
|  | Cancer | yes | no |
| **Physical illness child** | Hospital | >1 hospitalisation | no |
|  | Medical condition | yes | no |
|  | Physical disability | yes | no |
| **Intimate partner violence** | Partner used physical force such as pushing, slapping, hitting, holding them down, punching, strangling, beating them up, hitting them with an object | often (can distinguish under/over 18yrs) | yes (asked at 21yrs) |
|  | Partner has pressured / physically forced them into kissing, touching, something else / sexual intercourse | often (can distinguish under/over 18yrs) | yes (asked at 21yrs) |
|  | Behaviour partner has ever made them feel scared or frightened | often (can distinguish under/over 18yrs) | yes (asked at 21yrs) |
|  | Someone they have been out with has intentionally slapped/ kicked/ pushed/ grabbed/ shoved/ thrown something at/ hit/ used another form of violence on them | yes | no |
| **Financial difficulties** | Difficulty in affording food/heating | Very difficult | no |
|  | Became homeless | yes, affected them | yes, the reported age can be retrospective (where applicable in this row, marked by 'Split into' certain ages) |
| **Social support child** | Happy with number of friends | unhappy / no friends | no |
|  | Friends understand / support them | Not at all /Hardly ever or never | no |
|  | Has friends | no | no |
|  | Number of friends | none | no |
| **Social support parents** | Someone to share feelings with | never / none | no |
| **Neighbourhood** | Happy living in neighbourhood | strongly disagree | no |
|  | Opinion of neighbourhood | Not at all good/ Bad area | no |
| **Bullying** | Overt bullying victim items including: personal belongings stolen, threatened/blackmailed, hit/beaten up | weekly | no |
|  | Relational bullying victim items including: do something didn't want to, told lies about child | weekly | no |
|  | Friends tried to get teenager to do things didn't want to / told lies about teenager | weekly | no |
|  | Young person has been directly/relationally bullied | weekly | no |
|  | Child has been bullied | all the time | no |
|  | Upset by name calling/exclusion from groups or bullying | Most days | no |
|  | Someone threatened/blackmailed teenager | weekly | no |
| **Parent-child bond** | Harmony of adult/child interaction | many conflicts | no |
|  | When growing up respondent felt loved | Rarely | yes (asked at 23yrs) |
|  | Close to parents | Not very close to either | no |
|  | Close to child | never / no | no |

### Imputation model

For statistical analyses using the ACE measures, we recommend using multiple imputation to address missingness as the differences in socioeconomic variables suggest data is not missing-at-random (Supplemental Table 2). It is important to include auxiliary variables to the imputation model that are associated with missingness and/or the adversity variables. For this reason, we included a range of demographic indicators that are associated with missingness in the ACE-score as well as adverse experiences during pregnancy or after 18 years. Supplemental Table 1 describes how each variable was handled in the imputation model. Note the use of passive imputation for the ACE count scores and categorical ACE score variables, which are therefore constructed from the imputed ACE constructs and not used as an auxiliary variable for the imputation of the individual ACE constructs.

*Supplemental Table 3 Description of the variables in the imputation model.*

| **Variable** | **Type of variable** | **Regression model to predict missing in this variable** | **How variable was entered when used to predict missing in other variables** |
| --- | --- | --- | --- |
| ACE count score 0-16yrs | continuous | Passive imputation sum 10 ACEs^2^ | n/a^3^ |
| Four level categorical ACE count score 0-16yrs | categorical (4) | Passive imputation split the sum of 10 ACEs into 0,1,2-3 and 4+ ACEs | n/a^3^ |
| physical abuse 0-16yrs | dichotomous | Logistic regression | dichotomous |
| sexual abuse 0-16yrs | dichotomous | Logistic regression | dichotomous |
| emotional abuse 0-16yrs | dichotomous | Logistic regression | dichotomous |
| emotional neglect 0-16yrs | dichotomous | Logistic regression | dichotomous |
| bullying 0-16yrs | dichotomous | Logistic regression | dichotomous |
| violence between parents 0-16yrs | dichotomous | Logistic regression | dichotomous |
| substance household 0-16yrs | dichotomous | Logistic regression | dichotomous |
| mental health problems or suicide 0-16yrs | dichotomous | Logistic regression | dichotomous |
| parent convicted offence 0-16yrs | dichotomous | Logistic regression | dichotomous |
| parental separation 0-16yrs | dichotomous | Logistic regression | dichotomous |
| extended ACE count score 0-16yrs | continuous | Passive imputation sum 19 ACEs^1^ | n/a^3^ |
| Four level categorical extended ACE count score 0-16yrs | categorical (4) | Passive imputation split the sum of 19 ACEs into 0-1,2,3-5 and 6+ ACEs | n/a^3^ |
| social class 0-16yrs | dichotomous | Logistic regression | dichotomous |
| financial difficulties 0-16yrs | dichotomous | Logistic regression | dichotomous |
| satisfaction with neighbourhood 0-16yrs | dichotomous | Logistic regression | dichotomous |
| social support of child 0-16yrs | dichotomous | Logistic regression | dichotomous |
| social support of parent 0-16yrs | dichotomous | Logistic regression | dichotomous |
| violence between child and partner 0-16yrs | dichotomous | Logistic regression | dichotomous |
| physical illness of the child 0-16yrs | dichotomous | Logistic regression | dichotomous |
| physical illness of a parent 0-16yrs | dichotomous | Logistic regression | dichotomous |
| parent-child bond 0-16yrs | dichotomous | Logistic regression | dichotomous |
| Gender | categorical (2) | categorical (2) | Gender |
| Household social class at 18wks gestation | categorical (6) | Polytomous (unordered) regression | 5 indicator variables |
| Ethnicity child | categorical (2) | Logistic regression | 1 indicator variables |
| Maternal age in years at delivery | continuous | Predictive mean matching | continuous |
| Home ownership mother during pregnancy | categorical (7) | Polytomous (unordered) regression | 6 indicator variables |
| Marital status mother during pregnancy | categorical (6) | Polytomous (unordered) regression | 5 indicator variables |
| Parity | continuous | Predictive mean matching | continuous |
| Self-reported highest educational level mother | categorical (5) | Polytomous (unordered) regression | 4 indicator variables |
| Mother-reported highest educational level partner | categorical (5) | Polytomous (unordered) regression | 4 indicator variables |
| Maternal depression score (EPDS) at 18 wks gestation | continuous | Predictive mean matching | continuous |
| Maternal depression score (EPDS) at 32 wks gestation | continuous | Predictive mean matching | continuous |
| Partner depression score (EPDS) at 18 wks gestation | continuous | Predictive mean matching | continuous |
| Birthweight child in grams | continuous | Predictive mean matching | continuous |
| Gestational age in weeks at delivery | continuous | Predictive mean matching | continuous |
| Maternal pre-pregnancy weight (Kg) | continuous | Predictive mean matching | continuous |
| Maternal pre-pregnancy BMI | continuous | Predictive mean matching | continuous |
| Self-reported highest educational level partner | categorical (5) | Polytomous (unordered) regression | 4 indicator variables |
| Partner-reported highest educational level mother | categorical (5) | Polytomous (unordered) regression | 4 indicator variables |
| Mother became homeless during pregnancy | categorical (2) | Logistic regression | 1 indicator variables |
| Difficulty affording food during pregnancy | categorical (4) | Polytomous (unordered) regression | 3 indicator variables |
| Difficulty affording heating during pregnancy | categorical (4) | Polytomous (unordered) regression | 3 indicator variables |
| Mother’s opinion of neighbourhood during pregnancy | categorical (4) | Polytomous (unordered) regression | 3 indicator variables |
| Partner convicted of an offence during pregnancy | categorical (2) | Logistic regression | 1 indicator variables |
| Partner separated since pregnancy | dichotomous | Logistic regression | dichotomous |
| Mother divorced since pregnancy | dichotomous | Logistic regression | dichotomous |
| Partner hard drug use during pregnancy | categorical (2) | Logistic regression | 1 indicator variables |
| Mother s partner was emotionally cruel when child was 18yrs | dichotomous | Logistic regression | Dichotomous |
| Antidepressant use by mother when child was 18yrs | dichotomous | Logistic regression | Dichotomous |
| Maternal depression score (EPDS) when child was 18yrs | continuous | Predictive mean matching | continuous |
| Mother separated from partner when child was 18yrs | dichotomous | Logistic regression | dichotomous |
| Maternal AUDIT score when child was 18yrs | continuous | Predictive mean matching | continuous |
| Paternal AUDIT score when child was 18yrs | continuous | Predictive mean matching | continuous |
| Partner of child used physical force when child was 18-21yrs | dichotomous | Logistic regression | dichotomous |
| Partner of child used more severe physical force when child was 18-21yrs | dichotomous | Logistic regression | dichotomous |
| Partner of child have pressured them into kissing/touching when child was 18-21yrs | dichotomous | Logistic regression | dichotomous |
| Partner of child physically forced them into kissing/touching when child was 18-21yrs | dichotomous | Logistic regression | dichotomous |
| Partner of child used pressured them into sexual intercourse when child was 18-21yrs | dichotomous | Logistic regression | dichotomous |
| Partner of child physically forced them into sexual intercourse when child was 18-21yrs | dichotomous | Logistic regression | dichotomous |
| Partner of child made them feel scared of frightened when child was 18-21yrs | dichotomous | Logistic regression | dichotomous |
| Maternal smoking during the 1st trimester of pregnancy | dichotomous | Logistic regression | dichotomous |
| Maternal smoking during the 2nd trimester of pregnancy | dichotomous | Logistic regression | dichotomous |
| Maternal smoking during the 3rd trimester of pregnancy (prospectively reported) | dichotomous | Logistic regression | dichotomous |
| Maternal smoking during the 3rd trimester of pregnancy (retrospectively reported) | dichotomous | Logistic regression | dichotomous |

^1^Formula used for the passive imputation of the extended ACE score: ~I(as.integer(ses)+as.integer(neighbourhood)+as.integer(physical_ill_par)+as.integer(emotional_abuse)+as.integer(physical_abuse)+as.integer(sexual_abuse)+as.integer(mental_suicide_household)+as.integer(physical_ill_child)+as.integer(bond)+as.integer(parental_separation)+as.integer(emotional_neglect)+as.integer(violence_household)+as.integer(bullying)+as.integer(social_support_child)+as.integer(substance_household)+as.integer(financial_difficulties)+as.integer(parent_convicted)+as.integer(social_support_parent)+as.integer(intimate_partner_violence))

^2^Formula used for the passive imputation of the ACE score: ~I(as.integer(emotional_abuse)+as.integer(physical_abuse)+as.integer(sexual_abuse)+as.integer(mental_suicide_household)+as.integer(parental_separation)+as.integer(emotional_neglect)+as.integer(violence_household)+as.integer(bullying)+as.integer(substance_household)+as.integer(parent_convicted))

^3^ None of the four ACE count score variables were used as a predictor of missingness for the other variables in the imputation model.

### Prevalence estimates for variables in imputation model for ACE_0-16yrs_

*Supplemental table 4 Prevalence estimates for the variables in the imputation model. Prevalence is shown in the 3598 participants who answered 50% of the questions for the ten classic ACEs (‘complete’) versus 8489 participants who answered less than 50% of the question on one of the ten classic ACEs (‘missing’).*

| **Variable** | **Level of categorical variable** | **Missing**  (answered less than 50% of the question on one of the ten classic ACEs) | **Complete** (answered 50% of the questions for the ten classic ACEs) | p-value difference complete versus missing |
| --- | --- | --- | --- | --- |
| ACE-score n=3598 (mean (sd)) | | n/a | 1.77 (1.54) | n/a |
| Categorical ACE-score (n (%)) | 0 | n/a | 801 (22.3) | n/a |
|  | 1 | n/a | 1034 (28.7) |  |
|  | 2_3 | n/a | 1263 (35.1) |  |
|  | 4+ | n/a | 500 (13.9) |  |
| physical abuse (n (%)) | non-exposed | 2481 (87.1) | 3005 (83.5) | <0.001 |
|  | exposed | 368 (12.9) | 593 (16.5) |  |
| sexual abuse (n (%)) | non-exposed | 5387 (97.6) | 3479 (96.7) | 0.017 |
|  | exposed | 135 (2.4) | 119 (3.3) |  |
| emotional abuse (n (%)) | non-exposed | 2644 (79.6) | 2941 (81.7) | 0.024 |
|  | exposed | 679 (20.4) | 657 (18.3) |  |
| emotional neglect (n (%)) | non-exposed | 1682 (79.4) | 2928 (81.4) | 0.075 |
|  | exposed | 436 (20.6) | 670 (18.6) |  |
| bullying (n (%)) | non-exposed | 2694 (77.6) | 2669 (74.2) | 0.001 |
|  | exposed | 779 (22.4) | 929 (25.8) |  |
| violence between parents (n (%)) | non-exposed | 2224 (78.8) | 2972 (82.6) | <0.001 |
|  | exposed | 597 (21.2) | 626 (17.4) |  |
| substance household (n (%)) | non-exposed | 3358 (89.0) | 3317 (92.2) | <0.001 |
|  | exposed | 415 (11.0) | 281 (7.8) |  |
| mental health problems or suicide (n (%)) | non-exposed | 2093 (55.3) | 2135 (59.3) | 0.001 |
|  | exposed | 1690 (44.7) | 1463 (40.7) |  |
| parent convicted offence (n (%)) | non-exposed | 3779 (93.1) | 3338 (92.8) | 0.579 |
|  | exposed | 279 (6.9) | 260 (7.2) |  |
| parental separation (n (%)) | non-exposed | 2100 (69.9) | 2834 (78.8) | <0.001 |
|  | exposed | 905 (30.1) | 764 (21.2) |  |
| Extended ACE-score n=1109 (mean (sd)) | | n/a | 3.00 (2.20) | n/a |
| Extended categorical ACE-score (n (%)) | 0-1 | n/a | 282 (25.4) | n/a |
|  | 2 | n/a | 257 (23.2) |  |
|  | 3-5 | n/a | 433 (39.0) |  |
|  | 6+ | n/a | 137 (12.4) |  |
| social class (n (%)) | non-exposed | 3234 (88.7) | 1835 (93.7) | <0.001 |
|  | exposed | 413 (11.3) | 123 (6.3) |  |
| financial difficulties (n (%)) | non-exposed | 3341 (82.6) | 3249 (90.7) | <0.001 |
|  | exposed | 704 (17.4) | 335 (9.3) |  |
| satisfaction with neighbourhood (n (%)) | non-exposed | 4738 (90.9) | 3280 (91.3) | 0.472 |
|  | exposed | 476 (9.1) | 311 (8.7) |  |
| social support of child (n (%)) | non-exposed | 2774 (88.8) | 3204 (89.5) | 0.398 |
|  | exposed | 349 (11.2) | 376 (10.5) |  |
| social support of parent (n (%)) | non-exposed | 3818 (87.7) | 3235 (90.4) | <0.001 |
|  | exposed | 537 (12.3) | 345 (9.6) |  |
| violence between child and partner (n (%)) | non-exposed | 1614 (87.3) | 1956 (90.8) | <0.001 |
|  | exposed | 235 (12.7) | 198 (9.2) |  |
| physical illness of the child (n (%)) | non-exposed | 5140 (90.0) | 3322 (92.7) | <0.001 |
|  | exposed | 569 (10.0) | 261 (7.3) |  |
| physical illness of a parent (n (%)) | non-exposed | 1877 (76.7) | 2583 (75.3) | 0.225 |
|  | exposed | 569 (23.3) | 846 (24.7) |  |
| parent-child bond (n (%)) | non-exposed | 2659 (81.0) | 2855 (80.2) | 0.408 |
|  | exposed | 623 (19.0) | 705 (19.8) |  |
| Gender (n (%)) | Male | 4526 (53.3) | 1688 (46.9) | <0.001 |
|  | Female | 3963 (46.7) | 1910 (53.1) |  |
| Household social class at 18wks gestation (n (%)) | I - Professional | 516 (7.1) | 527 (14.9) | <0.001 |
|  | II - Managerial and technical | 2549 (35.0) | 1646 (46.6) |  |
|  | IIINM - Skilled non-manual | 2406 (33.0) | 945 (26.7) |  |
|  | IIIM - Skilled manual | 1056 (14.5) | 297 (8.4) |  |
|  | IV - Partly skilled | 616 (8.4) | 102 (2.9) |  |
|  | V - Unskilled | 150 (2.1) | 18 (0.5) |  |
| Ethnicity child (n (%)) | White | 6973 (94.7) | 3466 (97.4) | <0.001 |
|  | Non-white | 393 (5.3) | 92 (2.6) |  |
| Home ownership mother during pregnancy (n (%)) | Mortgaged | 5384 (69.7) | 3108 (87.7) | <0.001 |
|  | Owned | 175 (2.3) | 65 (1.8) |  |
|  | Council rented | 1166 (15.1) | 134 (3.8) |  |
|  | Private furnished rental | 349 (4.5) | 78 (2.2) |  |
|  | Private unfurnished rental | 236 (3.1) | 61 (1.7) |  |
|  | Housing authority rented | 146 (1.9) | 20 (0.6) |  |
|  | Other | 272 (3.5) | 79 (2.2) |  |
| Marital status mother during pregnancy (n (%)) | Never married | 1576 (20.3) | 321 (9.0) | <0.001 |
|  | Widowed | 10 (0.1) | 4 (0.1) |  |
|  | Divorced | 340 (4.4) | 108 (3.0) |  |
|  | Separated | 133 (1.7) | 27 (0.8) |  |
|  | 1st marriage | 5186 (66.8) | 2877 (80.4) |  |
|  | Marriage 2 or 3 | 514 (6.6) | 240 (6.7) |  |
| Self-reported highest educational level mother (n (%)) | Certificate of Secondary Education (CSE) | 1678 (22.1) | 301 (8.4) | <0.001 |
|  | Vocational | 835 (11.0) | 233 (6.5) |  |
|  | O level | 2780 (36.6) | 1185 (33.2) |  |
|  | A level | 1536 (20.2) | 1081 (30.3) |  |
|  | Degree | 762 (10.0) | 769 (21.5) |  |
| Mother-reported highest educational level partner (n (%)) | CSE | 2126 (29.4) | 442 (12.5) | <0.001 |
|  | Vocational | 673 (9.3) | 223 (6.3) |  |
|  | O level | 1565 (21.6) | 765 (21.7) |  |
|  | A level | 1818 (25.1) | 1085 (30.7) |  |
|  | Degree | 1054 (14.6) | 1015 (28.8) |  |
| Self-reported highest educational level partner (n (%)) | CSE | 1335 (24.1) | 382 (11.7) | <0.001 |
|  | Vocational | 530 (9.6) | 193 (5.9) |  |
|  | O level | 1291 (23.3) | 716 (22.0) |  |
|  | A level | 1513 (27.3) | 1007 (30.9) |  |
|  | Degree | 864 (15.6) | 956 (29.4) |  |
| Partner-reported highest educational level mother (n (%)) | CSE | 1251 (23.3) | 332 (10.3) | <0.001 |
|  | Vocational | 570 (10.6) | 211 (6.6) |  |
|  | O level | 1852 (34.5) | 998 (31.1) |  |
|  | A level | 1090 (20.3) | 944 (29.4) |  |
|  | Degree | 603 (11.2) | 726 (22.6) |  |
| Mother became homeless during pregnancy (n (%)) | No | 7046 (97.6) | 3406 (99.0) | <0.001 |
|  | Yes | 175 (2.4) | 36 (1.0) |  |
| Difficulty affording food during pregnancy (n (%)) | Not difficult | 5404 (73.4) | 2963 (84.8) | <0.001 |
|  | Some difficulty | 1262 (17.1) | 397 (11.4) |  |
|  | Fairly difficult | 579 (7.9) | 116 (3.3) |  |
|  | Very difficult | 122 (1.7) | 17 (0.5) |  |
| Difficulty affording heating during pregnancy (n (%)) | Not difficult | 5090 (69.1) | 2829 (81.0) | <0.001 |
|  | Some difficulty | 1367 (18.6) | 475 (13.6) |  |
|  | Fairly difficult | 670 (9.1) | 141 (4.0) |  |
|  | Very difficult | 240 (3.3) | 48 (1.4) |  |
| Mother’s opinion of neighbourhood during pregnancy (n (%)) | Very good area | 3077 (40.4) | 1656 (47.4) | <0.001 |
|  | Fairly good area | 3904 (51.3) | 1703 (48.8) |  |
|  | Not very good area | 474 (6.2) | 112 (3.2) |  |
|  | Bad area | 158 (2.1) | 22 (0.6) |  |
| Partner convicted of an offence during pregnancy (n (%)) | No | 5533 (98.3) | 3270 (99.5) | <0.001 |
|  | Yes | 94 (1.7) | 17 (0.5) |  |
| Partner separated since pregnancy (n (%)) | No | 5379 (98.0) | 3226 (99.8) | <0.001 |
|  | Yes | 107 (2.0) | 6 (0.2) |  |
| Mother divorced since pregnancy (n (%)) | No | 6733 (95.5) | 3333 (99.2) | <0.001 |
|  | Yes | 316 (4.5) | 26 (0.8) |  |
| Partner hard drug use during pregnancy (n (%)) | No | 5315 (98.1) | 3148 (98.7) | 0.085 |
|  | Yes | 101 (1.9) | 43 (1.3) |  |
| Mother’s partner was emotionally cruel when child was 18yrs (n (%)) | No | 1495 (94.8) | 2456 (95.9) | 0.115 |
|  | Yes | 82 (5.2) | 105 (4.1) |  |
| Antidepressant use by mother when child was 18yrs (n (%)) | No | 1297 (86.9) | 2216 (90.2) | 0.002 |
|  | Yes | 196 (13.1) | 241 (9.8) |  |
| Mother separated from partner when child was 18yrs (n (%)) | No | 1489 (94.2) | 2472 (96.4) | 0.001 |
|  | Yes | 92 (5.8) | 92 (3.6) |  |
| Partner of child used physical force when child was 18-21yrs (n (%)) | No | 1033 (78.7) | 1594 (83.3) | 0.001 |
|  | Yes | 279 (21.3) | 320 (16.7) |  |
| Partner of child used more severe physical force when child was 18-21yrs (n (%)) | No | 1110 (84.7) | 1705 (89.2) | <0.001 |
|  | Yes | 200 (15.3) | 206 (10.8) |  |
| Partner of child have pressured them into kissing/touching when child was 18-21yrs (n (%)) | No | 1111 (84.9) | 1662 (86.7) | 0.162 |
|  | Yes | 197 (15.1) | 254 (13.3) |  |
| Partner of child physically forced them into kissing/touching when child was 18-21yrs (n (%)) | No | 1137 (87.0) | 1726 (90.3) | 0.004 |
|  | Yes | 170 (13.0) | 186 (9.7) |  |
| Partner of child used pressured them into sexual intercourse when child was 18-21yrs (n (%)) | No | 1091 (83.5) | 1638 (85.8) | 0.072 |
|  | Yes | 216 (16.5) | 270 (14.2) |  |
| Partner of child physically forced them into sexual intercourse when child was 18-21yrs (n (%)) | No | 1140 (87.6) | 1729 (90.6) | 0.009 |
|  | Yes | 161 (12.4) | 180 (9.4) |  |
| Partner of child made them feel scared of frightened when child was 18-21yrs (n (%)) | No | 1035 (79.6) | 1566 (82.8) | 0.022 |
|  | Yes | 266 (20.4) | 325 (17.2) |  |
| Maternal smoking during the 1st trimester of pregnancy (n (%)) | No | 5676 (72.7) | 3148 (87.9) | <0.001 |
|  | Yes | 2136 (27.3) | 433 (12.1) |  |
| Maternal smoking during the 2nd trimester of pregnancy (n (%)) | No | 6106 (78.2) | 3258 (91.0) | <0.001 |
|  | Yes | 1706 (21.8) | 323 (9.0) |  |
| Maternal smoking during the 3rd trimester of pregnancy (prospectively reported) (n (%)) | No | 5282 (76.4) | 2994 (90.7) | <0.001 |
|  | Yes | 1635 (23.6) | 308 (9.3) |  |
| Maternal smoking during the 3rd trimester of pregnancy (retrospectively reported) (n (%)) | No | 5725 (77.4) | 3234 (91.2) | <0.001 |
|  | Yes | 1673 (22.6) | 311 (8.8) |  |
| Maternal age in years at delivery n=11684 (mean (sd)) | | 27.83 (4.91) | 29.78 (4.20) | <0.001 |
| Parity n=11215 (mean (sd)) | | 0.88 (1.03) | 0.70 (0.85) | <0.001 |
| Maternal depression score (EPDS) at 18 wks gestation n=10466 (mean (sd)) | | 7.14 (4.85) | 6.00 (4.39) | <0.001 |
| Maternal depression score (EPDS) at 32 wks gestation n=10816 (mean (sd)) | | 7.26 (5.15) | 6.14 (4.57) | <0.001 |
| Partner depression score (EPDS) at 18 wks gestation n=8739 (mean (sd)) | | 4.33 (3.99) | 3.79 (3.57) | <0.001 |
| Birthweight child in grams n=11540 (mean (sd)) | | 3400.26 (560.55) | 3429.26 (531.70) | 0.009 |
| Gestational age in weeks at delivery n=11684 (mean (sd)) | | 39.44 (1.90) | 39.49 (1.77) | 0.177 |
| Maternal pre-pregnancy weight (Kg) n=10505 (mean (sd)) | | 61.89 (11.23) | 61.53 (9.98) | 0.106 |
| Maternal pre-pregnancy BMI n=10405 (mean (sd)) | | 23.05 (3.94) | 22.74 (3.47) | <0.001 |
| Maternal depression score (EPDS) when child was 18yrs n=4104 (mean (sd)) | | 8.12 (5.80) | 7.05 (5.16) | <0.001 |
| Maternal AUDIT score when child was 18yrs n=3478 (mean (sd)) | | 8.11 (3.03) | 8.03 (2.95) | 0.454 |
| Paternal AUDIT score when child was 18yrs n=1816 (mean (sd)) | | 9.24 (3.24) | 9.11 (2.96) | 0.408 |

### Gender differences ACE_0-16yrs_

*Supplemental table 5 Distributions of ACE measures in the imputation datasets and in observed data (i.e. without imputation) in male(n=6214) and female(n=5873).*

| **Variables in imputation model** | **Male** | | | **Female** | | | **p-value** Gender difference imputed data |
| --- | --- | --- | --- | --- | --- | --- | --- |
|  | **% data imputed** | **Distribution** Mean (SE) for continuous variables  % for categorical variables  In | | **% data imputed** | **Distribution** Mean (SE) for continuous variables  % for categorical variables  In | |  |
|  |  | imputed | observed (n=6214) |  | imputed | observed (n=5873) |  |
| ACE-score | 72.8 | 2.17 (0.02) | 1.77 (0.04) | 67.5 | 2.18 (0.02) | 1.76 (0.04) | 0.75 |
| Categorical ACE-score 0 | 72.8 | 16.9 | 20.8 | 67.5 | 17.9 | 23.6 | 0.46 |
| 1 |  | 25.8 | 30.2 |  | 24.5 | 27.4 |  |
| 2-3 |  | 36 | 34.8 |  | 36.2 | 35.4 |  |
| 4+ |  | 21.3 | 14.2 |  | 21.4 | 13.6 |  |
| physical abuse | 49.2 | 16.1 | 13.6 | 44 | 19.2 | 16.2 | <0.01 |
| sexual abuse | 25.5 | 2 | 1.1 | 23.6 | 5.4 | 4.5 | <0.01 |
| emotional abuse | 44.5 | 22.4 | 18.9 | 40.9 | 22.7 | 19.7 | 0.82 |
| emotional neglect | 56.5 | 24.3 | 21.4 | 48.7 | 19.8 | 17.5 | <0.01 |
| bullying | 44.3 | 27.8 | 27.1 | 38.5 | 22.6 | 21.3 | <0.01 |
| violence between parents | 47.4 | 23.8 | 18.6 | 46.4 | 24.5 | 19.5 | 0.56 |
| substance household | 39.3 | 13.8 | 9.7 | 38.7 | 13.5 | 9.1 | 0.73 |
| mental health problems or suicide | 39.8 | 46 | 41.6 | 38 | 48.1 | 43.9 | 0.09 |
| parent convicted offence | 37 | 9.1 | 6.8 | 36.3 | 9.6 | 7.3 | 0.51 |
| parental separation | 46.4 | 31.5 | 24.1 | 44.3 | 33 | 26.4 | 0.22 |
| Extended ACE-score | 91.5 | 3.64 (0.03) | 3.06 (0.09) | 90.2 | 3.52 (0.03) | 2.95 (0.09) | 0.06 |
| Extended categorical ACE-score 0-1 | 91.5 | 28.3 | 23.5 | 90.2 | 29.9 | 27.2 | 0.29 |
| 2 |  | 22.7 | 23.5 |  | 22.3 | 22.8 |  |
| 3-5 |  | 47.8 | 41.1 |  | 46.3 | 37.2 |  |
| 6+ |  | 1.3 | 11.9 |  | 1.6 | 12.8 |  |
| social class | 53.5 | 11.3 | 9.1 | 53.7 | 12 | 10 | 0.47 |
| financial difficulties | 36.8 | 18.7 | 13.7 | 37 | 18 | 13.6 | 0.53 |
| satisfaction with neighbourhood | 28.2 | 10.5 | 8.4 | 26 | 11.1 | 9.5 | 0.36 |
| social support of child | 47.4 | 14.9 | 13.3 | 41.5 | 10.1 | 8.5 | <0.01 |
| social support of parent | 34.6 | 13.4 | 10.6 | 34.1 | 14.2 | 11.7 | 0.35 |
| violence between child and partner | 68.8 | 15.5 | 12.5 | 64.8 | 12.3 | 9.2 | <0.01 |
| physical illness of the child | 23.1 | 12.3 | 11.2 | 23.2 | 7.5 | 6.5 | <0.01 |
| physical illness of a parent | 51.8 | 27.1 | 23.5 | 50.9 | 27.1 | 24.7 | 0.96 |
| parent-child bond | 44.1 | 23.4 | 20 | 42.6 | 21.7 | 18.8 | 0.19 |

### Social class differences ACE_0-16yrs_

*Supplemental table 6 Distributions of the ACE measures in the imputation datasets and in observed data (i.e. without imputation) in low (= class V Unskilled) versus other social class (= class I Professional to class IV Partly skilled).*

| **Variables in imputation model** | **Social class V (unskilled)** | | | **Social class I (professional) to IV (partly skilled)** | | | **p-value** Social class difference imputed data |
| --- | --- | --- | --- | --- | --- | --- | --- |
|  | **% data imputed** | **Distribution** Mean (SE) for continuous variables  % for categorical variables  In | | **% data imputed** | **Distribution** Mean (SE) for continuous variables  % for categorical variables  In | |  |
|  |  | imputed | observed (n=168) |  | imputed | observed (n=10660) |  |
| ACE-score | 89.3 | 3.29 (0.14) | 2.28 (0.51) | 67 | 2.16 (0.02) | 1.76 (0.03) | <0.01 |
| Categorical ACE-score 0 | 89.3 | 6.6 | 16.7 | 67 | 17.6 | 22.5 | <0.01 |
| 1 |  | 15.3 | 27.8 |  | 25.4 | 28.8 |  |
| 2-3 |  | 36.2 | 38.9 |  | 36.1 | 34.9 |  |
| 4+ |  | 41.8 | 16.7 |  | 21 | 13.8 |  |
| physical abuse | 73.2 | 31 | 26.7 | 41.7 | 17.3 | 14.6 | 0.02 |
| sexual abuse | 33.3 | 11.5 | 8.9 | 20.3 | 3.5 | 2.6 | <0.01 |
| emotional abuse | 71.4 | 36.4 | 29.2 | 37.4 | 22.3 | 19.2 | 0.04 |
| emotional neglect | 73.2 | 32 | 26.7 | 51.4 | 21.9 | 19.2 | 0.12 |
| bullying | 58.3 | 28.3 | 27.1 | 40.3 | 25.2 | 24.4 | 0.56 |
| violence between parents | 70.2 | 42.4 | 32 | 43.3 | 23.8 | 18.7 | <0.01 |
| substance household | 64.3 | 21.5 | 10 | 33.5 | 13.5 | 9.3 | 0.18 |
| mental health problems or suicide | 62.5 | 59.1 | 49.2 | 33.5 | 46.8 | 42.5 | 0.02 |
| parent convicted offence | 60.7 | 16.9 | 10.6 | 31.2 | 9.2 | 6.9 | 0.08 |
| parental separation | 70.8 | 49.9 | 38.8 | 40.3 | 31.9 | 25 | <0.01 |
| Extended ACE-score | 97 | 5.79 (0.21) | 5.4 (1.47) | 89.8 | 3.54 (0.02) | 2.99 (0.07) | <0.01 |
| Extended categorical ACE-score 0-1 | 97 | 11.5 | 0 | 89.8 | 29.3 | 25.4 | <0.01 |
| 2 |  | 15.7 | 0 |  | 22.5 | 23.5 |  |
| 3-5 |  | 71.9 | 80 |  | 46.8 | 38.8 |  |
| 6+ |  | 0.9 | 20 |  | 1.4 | 12.3 |  |
| social class | 42.9 | 51.6 | 49 | 50.5 | 10.9 | 8.7 | <0.01 |
| financial difficulties | 61.3 | 28.2 | 16.9 | 31.4 | 18.2 | 13.2 | 0.06 |
| satisfaction with neighbourhood | 42.9 | 14.1 | 9.4 | 23.9 | 10.7 | 8.5 | 0.3 |
| social support of child | 60.1 | 18.6 | 16.4 | 43.6 | 12.5 | 10.5 | 0.14 |
| social support of parent | 56.5 | 27 | 20.5 | 28.8 | 13.5 | 10.8 | <0.01 |
| violence between child and partner | 73.8 | 22.7 | 18.2 | 66.4 | 13.7 | 10.4 | 0.14 |
| physical illness of the child | 29.8 | 16.2 | 13.6 | 18.8 | 9.8 | 8.7 | 0.06 |
| physical illness of a parent | 81.5 | 34.8 | 29 | 46.5 | 26.9 | 24 | 0.27 |
| parent-child bond | 68.5 | 37 | 30.2 | 38.2 | 22.3 | 19.3 | <0.01 |

### Maternal education differences ACE_0-16yrs_

*Supplemental table 7 Distributions of the ACE measures in the imputation datasets and in observed data (i.e. without imputation) in children from highly educated mothers (= degree) versus lower (= CSE, vocational, O or A-level). The adversity related variables are in grey, sociodemographic indicators in white.*

| **Variables in imputation model** | **Mother obtained at least a degree (or higher education level)** | | | **Mother obtained certificate of secondary education (CSE), vocational qualification, O-level or A-level** | | | **p-value** Maternal education difference imputed data |
| --- | --- | --- | --- | --- | --- | --- | --- |
|  | **% data imputed** | **Distribution** Mean (SE) for continuous variables  % for categorical variables  In | | **% data imputed** | **Distribution** Mean (SE) for continuous variables  % for categorical variables  In | |  |
|  |  | imputed | observed (n=1531) |  | imputed | observed (n=9629) |  |
| ACE-score | 49.8 | 1.8 (0.04) | 1.63 (0.05) | 70.9 | 2.24 (0.02) | 1.8 (0.03) | <0.01 |
| Categorical ACE-score 0 | 49.8 | 22.3 | 24.8 | 70.9 | 16.6 | 21.5 | <0.01 |
| 1 |  | 27.6 | 28.2 |  | 24.8 | 29 |  |
| 2-3 |  | 35.6 | 35.2 |  | 36.2 | 35 |  |
| 4+ |  | 14.6 | 11.7 |  | 22.4 | 14.4 |  |
| physical abuse | 24.8 | 17.8 | 16.1 | 46 | 17.5 | 14.7 | 0.83 |
| sexual abuse | 11.7 | 2.9 | 2.3 | 21.7 | 3.8 | 2.9 | 0.12 |
| emotional abuse | 22.6 | 21.6 | 19.3 | 41.5 | 22.7 | 19.4 | 0.43 |
| emotional neglect | 36.9 | 15.2 | 14.3 | 54.6 | 23.2 | 20.3 | <0.01 |
| bullying | 29.3 | 24.8 | 24.2 | 43.2 | 25.3 | 24.3 | 0.72 |
| violence between parents | 27.2 | 17.4 | 15.1 | 47.4 | 25.2 | 19.7 | <0.01 |
| substance household | 19.3 | 10.5 | 8.3 | 37.4 | 14.2 | 9.8 | <0.01 |
| mental health problems or suicide | 18.6 | 41 | 38.7 | 37.5 | 48 | 43.4 | <0.01 |
| parent convicted offence | 17.7 | 8.1 | 7.1 | 34.8 | 9.6 | 7 | 0.13 |
| parental separation | 24.3 | 21 | 18 | 44.5 | 34 | 26.9 | <0.01 |
| Extended ACE-score | 88.6 | 2.79 (0.05) | 2.65 (0.14) | 90.4 | 3.71 (0.03) | 3.07 (0.07) | <0.01 |
| Extended categorical ACE-score 0-1 | 88.6 | 34.3 | 26.9 | 90.4 | 28.1 | 25.2 | <0.01 |
| 2 |  | 24.5 | 28.6 |  | 22.1 | 22 |  |
| 3-5 |  | 40.4 | 37.1 |  | 48.3 | 39.5 |  |
| 6+ |  | 0.9 | 7.4 |  | 1.5 | 13.3 |  |
| social class | 57.4 | 4 | 0.8 | 49.7 | 12.9 | 10.6 | <0.01 |
| financial difficulties | 18.6 | 7.7 | 5.9 | 35.1 | 20 | 15 | <0.01 |
| satisfaction with neighbourhood | 13.8 | 6.8 | 5.8 | 26.1 | 11.4 | 9.4 | <0.01 |
| social support of child | 32.9 | 9.8 | 8.7 | 46.3 | 13 | 10.9 | <0.01 |
| social support of parent | 16.1 | 8.6 | 7.4 | 32.3 | 14.6 | 11.8 | <0.01 |
| violence between child and partner | 65.7 | 10.3 | 8 | 67 | 14.5 | 11.2 | 0.01 |
| physical illness of the child | 12.5 | 6.9 | 6.4 | 19.9 | 10.4 | 9.2 | <0.01 |
| physical illness of a parent | 30.5 | 24.3 | 22.6 | 50.9 | 27.5 | 24.2 | 0.04 |
| parent-child bond | 21.9 | 20.7 | 19.3 | 42.6 | 22.8 | 19.5 | 0.11 |

### Correlation between ACE constructs


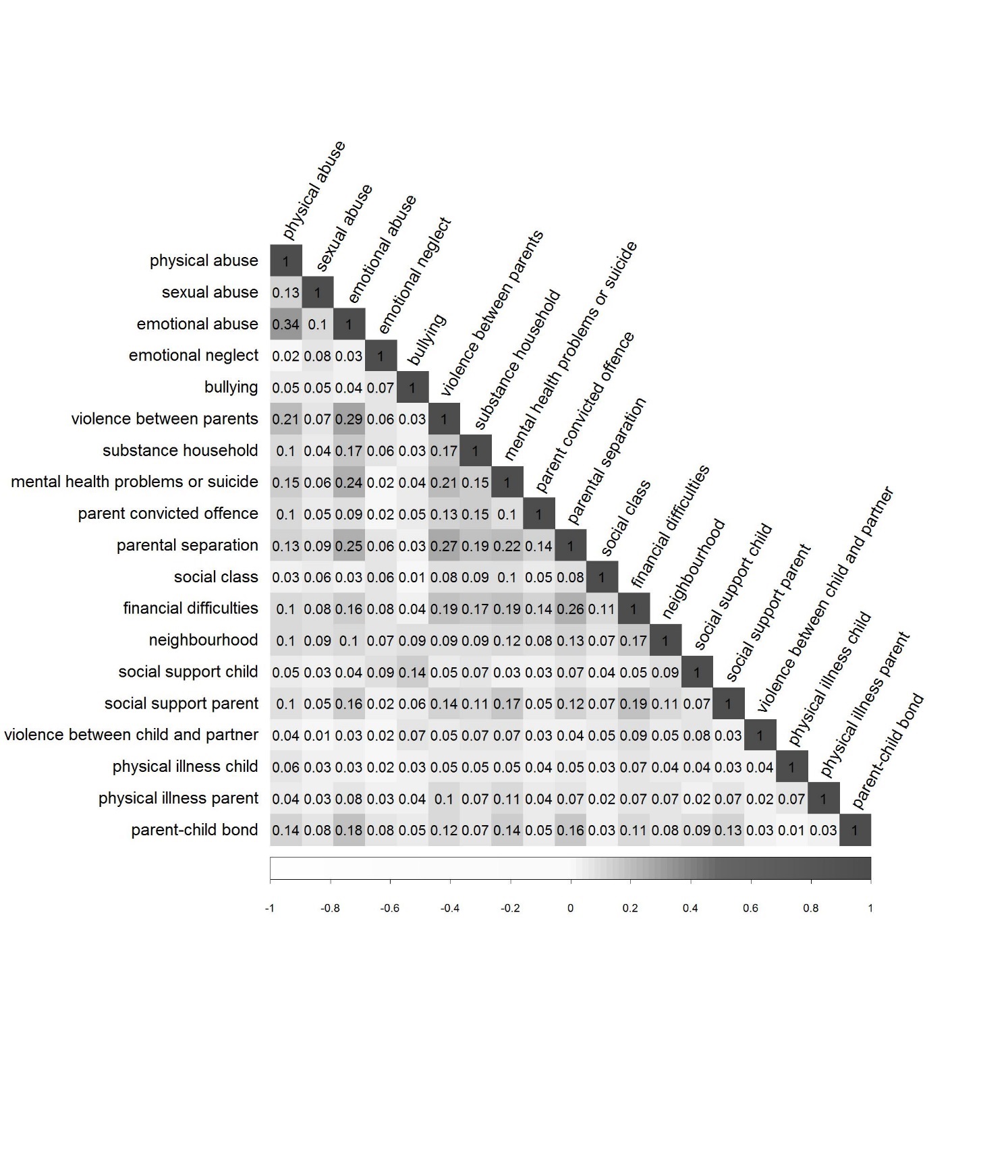


*Supplemental Figure 1 Correlation (Cramér's V based on chi squared) between the ACE measures for exposure between 0-16 years in ALSPAC. The shading indicates the strength of the correlation from low (white) to high (dark grey).*

### ACE questions

*Supplemental table 1 The alspac variables used to derive the adverse childhood experience category (ACE) constructs.*

| **id** | **Description** | **reported** | **retrospective** | **ACE** | **Age reported** | **Start** time period | **End** time period |
| --- | --- | --- | --- | --- | --- | --- | --- |
| ccc290 | Frequency child has been bullied | Child | no | bullying | 97m | 8 | 8 |
| f8fp141 | Personal belongings stolen | Child | no | bullying | 8.5yrs | 8.5 | 8.5 |
| f8fp151 | Threatened/blackmailed | Child | no | bullying | 8.5yrs | 8.5 | 8.5 |
| f8fp161 | Hit/beaten up | Child | no | bullying | 8.5yrs | 8.5 | 8.5 |
| f8fp341 | Got to do something didn't want to | Child | no | bullying | 8.5yrs | 8.5 | 8.5 |
| f8fp351 | Told lies about | Child | no | bullying | 8.5yrs | 8.5 | 8.5 |
| fdfp141 | Personal belongings stolen | Child | no | bullying | 10+yrs | 10 | 10 |
| fdfp151 | Threatened/blackmailed | Child | no | bullying | 10+yrs | 10 | 10 |
| fdfp161 | Hit/beaten up | Child | no | bullying | 10+yrs | 10 | 10 |
| fdfp341 | Got to do something didn't want to | Child | no | bullying | 10+yrs | 10 | 10 |
| fdfp351 | Told lies about | Child | no | bullying | 10+yrs | 10 | 10 |
| ff6011 | Someone threatened/blackmailed teenager | Child | no | bullying | 12.5yrs | 12.5 | 12.5 |
| ff6211 | Friends tried to get teenager to do things didnt want to do | Child | no | bullying | 12.5yrs | 12.5 | 12.5 |
| ff6221 | Friends told lies about teenager | Child | no | bullying | 12.5yrs | 12.5 | 12.5 |
| fh8332 | YPs friends put YP down in front of others | Child | no | bullying | 15.5yrs | 15.5 | 15.5 |
| fh8334 | YPs friends put pressure on YP to do things they don't want to do | Child | no | bullying | 15.5yrs | 15.5 | 15.5 |
| ccxa210 | During the last school year YP has been upset by name calling/exclusion from groups or bullying | Child | no | bullying | year 11 | 16 | 16 |
| fjpc4000 | YP has been directly bullied in last 6 mths | Child | no | bullying | 17.5yrs | 17.5 | 17.5 |
| fjpc4100 | YP has been relationally bullied in last 6 mths | Child | no | bullying | 17.5yrs | 17.5 | 17.5 |
| b608 | PTNR was EMOT cruel to CH since PREG | Parent | no | emotional abuse | 18w gest | -1 | -1 |
| f257 | PTNR EMOT cruel to CHDR >CH born | Parent | no | emotional abuse | 8m | 0 | 0.67 |
| f258 | MUM EMOT cruel to CHDR >CH born | Parent | no | emotional abuse | 8m | 0 | 0.67 |
| pd257 | Ptnr Emotionally Cruel To Children | Parent | no | emotional abuse | 8m | 0 | 0.67 |
| pd258 | Self Emotionally Cruel To Children | Parent | no | emotional abuse | 8m | 0 | 0.67 |
| ypb8000 | Frequency adult in family shouted at respondent before age of 11 | Child | yes | emotional abuse | 22yrs | 0 | 11 |
| ypb8001 | Frequency adult in family said hurtful or insulting things to respondent before age of 11 | Child | yes | emotional abuse | 22yrs | 0 | 11 |
| ypc1812 | When growing up respondent felt that someone in their family hated them | Child | yes | emotional abuse | 23yrs | 0 | 16 |
| pc236 | PTNR EMOT cruel to CH | Parent | no | emotional abuse | 8w | 0.16 | 0.16 |
| g337 | Partner emotionally cruel to children >CH8MTHs | Parent | no | emotional abuse | 1yrs9m | 0.67 | 2 |
| g338 | Mum emotionally cruel to children >CH18MTHs | Parent | no | emotional abuse | 1yrs9m | 0.67 | 2 |
| pe337 | Partner Emotionally Cruel To Child | Parent | no | emotional abuse | 1yrs9m | 0.67 | 2 |
| pe338 | Self Emotionally Cruel To Child | Parent | no | emotional abuse | 1yrs9m | 0.67 | 2 |
| h247 | Whether partner was emotionally cruel to children since study child was 18 months old and effect this had | Parent | no | emotional abuse | 2yrs9m | 1.5 | 3 |
| h248 | Whether mum was emotionally cruel to children since study child was 18 months old and effect this had | Parent | no | emotional abuse | 2yrs9m | 1.5 | 3 |
| pf5036 | Partner's partner was emotionally cruel to their children since study child was 18 months old | Parent | no | emotional abuse | 2yrs9m | 1.5 | 3 |
| pf5037 | Partner was emotionally cruel to their children since study child was 18 months old | Parent | no | emotional abuse | 2yrs9m | 1.5 | 3 |
| j337 | PTR Emotional Cruel to CDRN> CH 30 MTHs | Parent | no | emotional abuse | 3yrs11m | 2.5 | 4 |
| j338 | MUM Emotional Cruel to CDRN> CH 30 MTHs | Parent | no | emotional abuse | 3yrs11m | 2.5 | 4 |
| pg3036 | Partner's emotional cruelty towards children affected partner since child was 2.5 years old | Parent | no | emotional abuse | 3yrs11m | 2.5 | 4 |
| pg3037 | Partner's emotional cruelty towards children affected partner since child was 2.5 years old | Parent | no | emotional abuse | 3yrs11m | 2.5 | 4 |
| k4037 | Mothers partner was emotionally cruel to children in past year | Parent | no | emotional abuse | 5yrs1m | 4 | 5 |
| k4038 | Mother was emotionally cruel to children in past year | Parent | no | emotional abuse | 5yrs1m | 4 | 5 |
| ph4037 | Respondent's assessment of how much their partner being emotionally cruel to the children in the last year has affected them | Parent | no | emotional abuse | 5yrs1m | 4 | 5 |
| ph4038 | Respondent's assessment of how much being emotionally cruel to their children in the last year has affected them | Parent | no | emotional abuse | 5yrs1m | 4 | 5 |
| l4037 | Respondent's partner was emotionally cruel to respondent's children since study child's 5th birthday | Parent | no | emotional abuse | 6yrs1m | 5 | 6 |
| l4038 | Respondent was emotionally cruel to their children since study child's 5th birthday | Parent | no | emotional abuse | 6yrs1m | 5 | 6 |
| pj4037 | Respondent's assessment of how much partner's emotional cruelty to children since study child's 5th birthday has affected them | Parent | no | emotional abuse | 6yrs1m | 5 | 6 |
| pj4038 | Respondent's assessment of how much being emotionally cruel to children since study child's 5th birthday has affected them | Parent | no | emotional abuse | 6yrs1m | 5 | 6 |
| p2037 | Mother's husband/partner was emotionally cruel to her children since the study child's 6th birthday | Parent | yes | emotional abuse | 9yrs2m | 6 | 7 |
| p2038 | Mother was emotionally cruel to her children since the study child's 6th birthday | Parent | yes | emotional abuse | 9yrs2m | 6 | 7 |
| pm2037 | Father's wife/partner was emotionally cruel to his children since the study child's 6th birthday | Parent | yes | emotional abuse | 9yrs2m | 6 | 7 |
| pm2038 | Father was emotionally cruel to his children since the study child's 6th birthday | Parent | yes | emotional abuse | 9yrs2m | 6 | 7 |
| p2037_dup | Mother's husband/partner was emotionally cruel to her children since the study child's 6th birthday | Parent | no | emotional abuse | 9yrs2m | 8 | 9 |
| p2038_dup | Mother was emotionally cruel to her children since the study child's 6th birthday | Parent | no | emotional abuse | 9yrs2m | 8 | 9 |
| pm2037_dup | Father's wife/partner was emotionally cruel to his children since the study child's 6th birthday | Parent | no | emotional abuse | 9yrs2m | 8 | 9 |
| pm2038_dup | Father was emotionally cruel to his children since the study child's 6th birthday | Parent | no | emotional abuse | 9yrs2m | 8 | 9 |
| pp5037 | Respondent's wife/partner has been emotionally cruel to their children since the study child's 9th birthday | Parent | yes | emotional abuse | 11yrs2m | 9 | 10 |
| pp5038 | Respondent has been emotionally cruel to their children since the study child's 9th birthday | Parent | yes | emotional abuse | 11yrs2m | 9 | 10 |
| r5037 | Respondent's husband/partner has been emotionally cruel to their children since study child's 9th birthday | Parent | yes | emotional abuse | 11yrs2m | 9 | 10 |
| r5038 | Respondent has been emotionally cruel to their children since study child's 9th birthday | Parent | yes | emotional abuse | 11yrs2m | 9 | 10 |
| pp5037_dup | Respondent's wife/partner has been emotionally cruel to their children since the study child's 9th birthday | Parent | no | emotional abuse | 11yrs2m | 11 | 11 |
| pp5038_dup | Respondent has been emotionally cruel to their children since the study child's 9th birthday | Parent | no | emotional abuse | 11yrs2m | 11 | 11 |
| r5037_dup | Respondent's husband/partner has been emotionally cruel to their children since study child's 9th birthday | Parent | no | emotional abuse | 11yrs2m | 11 | 11 |
| r5038_dup | Respondent has been emotionally cruel to their children since study child's 9th birthday | Parent | no | emotional abuse | 11yrs2m | 11 | 11 |
| ypb8050 | Frequency adult in family shouted at respondent between ages of 11 and 17 | Child | yes | emotional abuse | 22yrs | 11 | 17 |
| ypb8051 | Frequency adult in family said hurtful or insulting things to respondent between ages of 11 and 17 | Child | yes | emotional abuse | 22yrs | 11 | 17 |
| t3336 | Respondent's partner was emotionally cruel to respondent's children in last year | Parent | no | emotional abuse | 2010 | 17.5 | 18.5 |
| t3337 | Respondent was emotionally cruel to own children in last year | Parent | no | emotional abuse | 2010 | 17.5 | 18.5 |
| fa3333 | In last year, partner was emotionally cruel to respondent's children | Parent | no | emotional abuse | 2011-2013 | 19.5 | 20.5 |
| fa3334 | In last year, was emotionally cruel to own children | Parent | no | emotional abuse | 2011-2013 | 19.5 | 20.5 |
| ypb8100 | Frequency someone in family made respondent feel important or special, before age of 11 | Child | yes | emotional neglect | 22yrs | 0 | 11 |
| ypc1814 | When growing up there was someone to take respondent to the doctor if needed | Child | yes | emotional neglect | 23yrs | 0 | 16 |
| ccc250 | Frequency child feels left out of things | Child | no | emotional neglect | 97m | 8 | 8 |
| ccf104 | Study child is understood by parent(s) | Child | no | emotional neglect | 116m | 9.5 | 9.5 |
| ypb8120 | Frequency someone in family made respondent feel important or special, between ages 11 and 17 | Child | yes | emotional neglect | 22yrs | 11 | 17 |
| ff5316 | Frequency carers ask teenager what happened in their free time | Child | no | emotional neglect | 12.5yrs | 12.5 | 12.5 |
| ff5317 | Frequency past month carers started conversation about teenagers spare time | Child | no | emotional neglect | 12.5yrs | 12.5 | 12.5 |
| ff5318 | Frequency carers take time to listen, when teenager talks about what happened in their free time | Child | no | emotional neglect | 12.5yrs | 12.5 | 12.5 |
| ff5331 | Frequency carers know who teenagers friends are, outside of school | Child | no | emotional neglect | 12.5yrs | 12.5 | 12.5 |
| ff5346 | Frequency carers ask teenager what has happened at school, on normal school day | Child | no | emotional neglect | 12.5yrs | 12.5 | 12.5 |
| fg7116 | Frequency carers ask teenager what happened in free time | Child | no | emotional neglect | 13.5yrs | 13.5 | 13.5 |
| fg7118 | Frequency carers take time to listen, when teenager talks about what happened in free time | Child | no | emotional neglect | 13.5yrs | 13.5 | 13.5 |
| fg7131 | Frequency carers know who teenagers friends are, outside of school | Child | no | emotional neglect | 13.5yrs | 13.5 | 13.5 |
| fg7146 | Frequency carers ask teenager what has happened at school, on normal school day | Child | no | emotional neglect | 13.5yrs | 13.5 | 13.5 |
| fh9819 | Frequency carer asks YP what they did in their free time | Child | no | emotional neglect | 15.5yrs | 15.5 | 15.5 |
| fh9820 | Frequency carer started conversation about YPs free time, in last month | Child | no | emotional neglect | 15.5yrs | 15.5 | 15.5 |
| fh9821 | Frequency carer listens to YP, when they talk about what they they did in their free time | Child | no | emotional neglect | 15.5yrs | 15.5 | 15.5 |
| fh9838 | Frequency carer asks YP about what happened at school on normal school day | Child | no | emotional neglect | 15.5yrs | 15.5 | 15.5 |
| ccxa240 | Frequency over last term parents have asked YP how they are getting on with different subjects at school | Child | no | emotional neglect | year 11 | 16 | 16 |
| txa220 | Frequency in recent months parent/carer/other adult in household has talked to study child about study child's experiences at school | Parent | no | emotional neglect | school year 11/192m | 16 | 16 |
| txa221 | Frequency in recent months parent/carer/other adult in household has talked to study child about study child's friends | Parent | no | emotional neglect | school year 11/192m | 16 | 16 |
| txa222 | Frequency in recent months parent/carer/other adult in household has talked to study child about things that are troubling study child | Parent | no | emotional neglect | school year 11/192m | 16 | 16 |
| fjpc2100 | How easy YP finds it to discuss their problems with anyone in their family | Child | no | emotional neglect | 17.5yrs | 17.5 | 17.5 |
| b593 | Became homeless since PREG | Parent | no | financial difficulties | 18w gest | -1 | -1 |
| c472 | Homeless this PREG | Parent | no | financial difficulties | 32w gest | -1 | -1 |
| c520 | Difficulty in affording food | Parent | no | financial difficulties | 32w gest | -1 | -1 |
| c522 | Difficulty in affording heating | Parent | no | financial difficulties | 32w gest | -1 | -1 |
| pb183 | Became homeless since PTNR PREG | Parent | no | financial difficulties | 18w gest | -1 | -1 |
| f243 | Became homeless >CH born | Parent | no | financial difficulties | 8m | 0 | 0.67 |
| pd243 | Became Homeless Since Baby Born | Parent | no | financial difficulties | 8m | 0 | 0.67 |
| e423 | Became homeless since PREG | Parent | no | financial difficulties | 8w | -1 | 0.16 |
| pc223 | Became homeless since MID PREG | Parent | no | financial difficulties | 8w | -1 | 0.16 |
| f800 | Difficulty affording food | Parent | no | financial difficulties | 8m | 0.67 | 0.67 |
| f802 | Difficulty affording heating | Parent | no | financial difficulties | 8m | 0.67 | 0.67 |
| pd680 | Difficulty Affording Food | Parent | no | financial difficulties | 8m | 0.67 | 0.67 |
| pd682 | Difficulty Affording Heating | Parent | no | financial difficulties | 8m | 0.67 | 0.67 |
| g323 | Mum became homeless >CH8MTHs | Parent | no | financial difficulties | 1yrs9m | 0.67 | 2 |
| pe323 | Became Homeless | Parent | no | financial difficulties | 1yrs9m | 0.67 | 2 |
| h233 | Whether mum became homeless since study child was 18 months old and effect this had | Parent | no | financial difficulties | 2yrs9m | 1.5 | 3 |
| pf5023 | Partner became homeless since study child was 18 months old | Parent | no | financial difficulties | 2yrs9m | 1.5 | 3 |
| g835 | Difficulty affording food | Parent | no | financial difficulties | 1yrs9m | 2 | 2 |
| g837 | Difficulty affording heating | Parent | no | financial difficulties | 1yrs9m | 2 | 2 |
| pe460 | Difficulty Affording Food | Parent | no | financial difficulties | 1yrs9m | 2 | 2 |
| pe462 | Difficulty Affording Heating | Parent | no | financial difficulties | 1yrs9m | 2 | 2 |
| j323 | MUM Became Homeless> CH 30 MTHs | Parent | no | financial difficulties | 3yrs11m | 2.5 | 4 |
| pg3023 | Degree to which becoming homeless affected partner since child was 2.5 years old | Parent | no | financial difficulties | 3yrs11m | 2.5 | 4 |
| h730 | Mums difficulty affording food now | Parent | no | financial difficulties | 2yrs9m | 3 | 3 |
| h732 | Mums difficulty affording heating now | Parent | no | financial difficulties | 2yrs9m | 3 | 3 |
| pf7110 | Partner finds it difficult at the moment to afford food | Parent | no | financial difficulties | 2yrs9m | 3 | 3 |
| pf7112 | Partner finds it difficult at the moment to afford heating | Parent | no | financial difficulties | 2yrs9m | 3 | 3 |
| k4023 | Mother became homeless in past year | Parent | no | financial difficulties | 5yrs1m | 4 | 5 |
| ph4023 | Respondent's assessment of how much becoming homeless in the last year has affected them | Parent | no | financial difficulties | 5yrs1m | 4 | 5 |
| k6200 | Mother finds it difficult to afford food | Parent | no | financial difficulties | 5yrs1m | 5 | 5 |
| k6202 | Mother finds it difficult to afford heating | Parent | no | financial difficulties | 5yrs1m | 5 | 5 |
| ph6200 | Ease with which respondent can afford food | Parent | no | financial difficulties | 5yrs1m | 5 | 5 |
| ph6202 | Ease with which respondent can afford heating | Parent | no | financial difficulties | 5yrs1m | 5 | 5 |
| l4023 | Respondent became homeless since study child's 5th birthday | Parent | no | financial difficulties | 6yrs1m | 5 | 6 |
| pj4023 | Respondent's assessment of how much becoming homeless since study child's 5th birthday has affected them | Parent | no | financial difficulties | 6yrs1m | 5 | 6 |
| p2023 | Mother became homeless since the study child's 6th birthday | Parent | yes | financial difficulties | 9yrs2m | 6 | 7 |
| pm2023 | Father became homeless since the study child's 6th birthday | Parent | yes | financial difficulties | 9yrs2m | 6 | 7 |
| m5170 | Degree of difficulty for mother to afford food | Parent | no | financial difficulties | 7yrs1m | 7 | 7 |
| m5172 | Degree of difficulty for mother to afford heating | Parent | no | financial difficulties | 7yrs1m | 7 | 7 |
| pk5170 | Degree to which partner finds it difficult to afford food | Parent | no | financial difficulties | 7yrs1m | 7 | 7 |
| pk5172 | Degree to which partner finds it difficult to afford heating | Parent | no | financial difficulties | 7yrs1m | 7 | 7 |
| p2023_dup | Mother became homeless since the study child's 6th birthday | Parent | no | financial difficulties | 9yrs2m | 8 | 9 |
| pm2023_dup | Father became homeless since the study child's 6th birthday | Parent | no | financial difficulties | 9yrs2m | 8 | 9 |
| pp5023 | Respondent has become homeless since the study child's 9th birthday | Parent | yes | financial difficulties | 11yrs2m | 9 | 10 |
| r5023 | Respondent became homeless since study child's 9th birthday | Parent | yes | financial difficulties | 11yrs2m | 9 | 10 |
| pp5023_dup | Respondent has become homeless since the study child's 9th birthday | Parent | no | financial difficulties | 11yrs2m | 11 | 11 |
| r5023_dup | Respondent became homeless since study child's 9th birthday | Parent | no | financial difficulties | 11yrs2m | 11 | 11 |
| pp9000 | Difficulty respondent has paying for food | Parent | no | financial difficulties | 11yrs2m | 11 | 11 |
| pp9002 | Difficulty respondent has paying for heating | Parent | no | financial difficulties | 11yrs2m | 11 | 11 |
| r9000 | Difficulty respondent has paying for food at the moment | Parent | no | financial difficulties | 11yrs2m | 11 | 11 |
| r9002 | Difficulty respondent has paying for heating at the moment | Parent | no | financial difficulties | 11yrs2m | 11 | 11 |
| t3322 | Respondent became homeless in last year | Parent | no | financial difficulties | 2010 | 17.5 | 18.5 |
| t1360 | Degree of difficulty respondent's household finds at the moment trying to afford food | Parent | no | financial difficulties | 2010 | 18.5 | 18.5 |
| t1362 | Degree of difficulty respondent's household finds at the moment trying to afford heating | Parent | no | financial difficulties | 2010 | 18.5 | 18.5 |
| fa3322 | In last year, became homeless | Parent | no | financial difficulties | 2011-2013 | 19.5 | 20.5 |
| fa1360 | Difficulty for household to afford food at the moment | Parent | no | financial difficulties | 2011-2013 | 20.5 | 20.5 |
| fa1362 | Difficulty for household to afford heating at the moment | Parent | no | financial difficulties | 2011-2013 | 20.5 | 20.5 |
| b370 | Edinburgh Postnatal Depression Score | Parent | no | mental health problems | 18w gest | -1 | -1 |
| c600 | EPDS in YP | Parent | no | mental health problems | 32w gest | -1 | -1 |
| pb260 | EPDS Score I | Parent | no | mental health problems | 18w gest | -1 | -1 |
| b106 | Medication for anxiety this PREG | Parent | no | mental health problems | 18w gest | -1 | -1 |
| b107 | Medication for anxiety in 1st 3 months | Parent | no | mental health problems | 18w gest | -1 | -1 |
| b122 | Medication for depression this PREG | Parent | no | mental health problems | 18w gest | -1 | -1 |
| b123 | Medication for depression in 1st 3 months | Parent | no | mental health problems | 18w gest | -1 | -1 |
| b597 | Attempted suicide since PREG | Parent | no | mental health problems | 18w gest | -1 | -1 |
| c093 | MEDTN for anxiety in last 3MTHS | Parent | no | mental health problems | 32w gest | -1 | -1 |
| c101 | MEDTN for depression in last 3MTHS | Parent | no | mental health problems | 32w gest | -1 | -1 |
| d152 | Had bulimia | Parent | no | mental health problems | 15w gest | -1 | -1 |
| d169 | Had schizophrenia | Parent | no | mental health problems | 15w gest | -1 | -1 |
| d170 | Had anorexia nervosa | Parent | no | mental health problems | 15w gest | -1 | -1 |
| pa172 | Had bulimia | Parent | no | mental health problems | 12w gest | -1 | -1 |
| pa189 | Had schizophrenia | Parent | no | mental health problems | 12w gest | -1 | -1 |
| pa190 | Had anorexia nervosa | Parent | no | mental health problems | 12w gest | -1 | -1 |
| pb187 | Attempted suicide since PTNR PREG | Parent | no | mental health problems | 18w gest | -1 | -1 |
| e326 | FREQ of anti-depressant use since birth | Parent | no | mental health problems | 8w | 0 | 0.16 |
| f063 | Anti-depressant use since CH born | Parent | no | mental health problems | 8m | 0 | 0.67 |
| f248 | Attempted suicide > CH born | Parent | no | mental health problems | 8m | 0 | 0.67 |
| f526 | PTNR had schizophrenia >CH born | Parent | no | mental health problems | 8m | 0 | 0.67 |
| pd063 | Used Pills for Depression Since Baby Born | Parent | no | mental health problems | 8m | 0 | 0.67 |
| pd248 | Attempted Suicide Since Baby Born | Parent | no | mental health problems | 8m | 0 | 0.67 |
| e391 | EPDS | Parent | no | mental health problems | 8w | 0.16 | 0.16 |
| pc102 | EPDS Score I | Parent | no | mental health problems | 8w | 0.16 | 0.16 |
| e427 | Attempted suicide since MID PREG | Parent | no | mental health problems | 8w | -1 | 0.16 |
| pc227 | Attempted suicide since MID PREG | Parent | no | mental health problems | 8w | -1 | 0.16 |
| f200 | Edinburgh Post-natal Depression Score | Parent | no | mental health problems | 8m | 0.67 | 0.67 |
| pd200 | Edinburgh Post-natal Depression Score | Parent | no | mental health problems | 8m | 0.67 | 0.67 |
| g049 | Mum had depression pills >CH8MTHs | Parent | no | mental health problems | 1yrs9m | 0.67 | 2 |
| g328 | Mum attempted suicide >CH8MTHs | Parent | no | mental health problems | 1yrs9m | 0.67 | 2 |
| g612 | Partner had schizophrenia >CH8MTHs | Parent | no | mental health problems | 1yrs9m | 0.67 | 2 |
| pe020 | Anxiety Since Child > 8 Months | Parent | no | mental health problems | 1yrs9m | 0.67 | 2 |
| pe064 | Taken Antidepressants CH > 8 Months | Parent | no | mental health problems | 1yrs9m | 0.67 | 2 |
| h039 | Frequency Mum has taken pills for depression since study child was 18 months old | Parent | no | mental health problems | 2yrs9m | 1.5 | 3 |
| h238 | Whether mum attempted suicide since study child was 18 months old and effect this had | Parent | no | mental health problems | 2yrs9m | 1.5 | 3 |
| h497 | Partner had schizophrenia since study child was 18 months old | Parent | no | mental health problems | 2yrs9m | 1.5 | 3 |
| pf5028 | Partner attempted suicide since study child was 18 months old | Parent | no | mental health problems | 2yrs9m | 1.5 | 3 |
| g290 | Edinburgh Post-natal Depression Score | Parent | no | mental health problems | 1yrs9m | 2 | 2 |
| pe290 | EPDS Score I | Parent | no | mental health problems | 1yrs9m | 2 | 2 |
| pe328 | Attempted Suicide | Parent | no | mental health problems | 1yrs9m | 2 | 2 |
| j328 | MUM Attempted Suicide> CH 30 MTHs | Parent | no | mental health problems | 3yrs11m | 2.5 | 4 |
| pg3028 | Degree to which attempted suicide affected partner since child was 2.5 years old | Parent | no | mental health problems | 3yrs11m | 2.5 | 4 |
| h200a | Edinburgh postnatal depression scale score (complete cases) | Parent | no | mental health problems | 2yrs9m | 3 | 3 |
| j044 | MUM took Depression Pills >1 YR | Parent | no | mental health problems | 3yrs11m | 3 | 4 |
| j615 | Partner had Schizophrenia> 1 YR | Parent | no | mental health problems | 3yrs11m | 3 | 4 |
| pg1034 | Frequency partner has taken pills for depression in the past year | Parent | no | mental health problems | 3yrs11m | 3 | 4 |
| k1020 | Mother had schizophrenia in past year | Parent | no | mental health problems | 5yrs1m | 4 | 5 |
| k1044 | Frequency mother had pills for depression in past year | Parent | no | mental health problems | 5yrs1m | 4 | 5 |
| k4028 | Mother attempted suicide in past year | Parent | no | mental health problems | 5yrs1m | 4 | 5 |
| ph1020 | Respondent had schizophrenia in the past year | Parent | no | mental health problems | 5yrs1m | 4 | 5 |
| ph1044 | Frequency in the past year respondent has taken pills for depression | Parent | no | mental health problems | 5yrs1m | 4 | 5 |
| ph4028 | Respondent's assessment of how much attempting suicide in the last year has affected them | Parent | no | mental health problems | 5yrs1m | 4 | 5 |
| l3020 | Respondent has had/continued to have schizophrenia since study child's 5th birthday | Parent | no | mental health problems | 6yrs1m | 5 | 6 |
| l3044 | Frequency respondent has taken pills for depression since study child's 5th birthday | Parent | no | mental health problems | 6yrs1m | 5 | 6 |
| l4028 | Respondent attempted suicide since study child's 5th birthday | Parent | no | mental health problems | 6yrs1m | 5 | 6 |
| l6031 | Respondent's partner has had schizophrenia since study child was 5 years old | Parent | no | mental health problems | 6yrs1m | 5 | 6 |
| pj3020 | Respondent has suffered from schizophrenia since child's 5th birthday | Parent | no | mental health problems | 6yrs1m | 5 | 6 |
| pj3044 | Respondent has taken pills for depression since child's 5th birthday | Parent | no | mental health problems | 6yrs1m | 5 | 6 |
| pj4028 | Respondent's assessment of how much attempting suicide since study child's 5th birthday has affected them | Parent | no | mental health problems | 6yrs1m | 5 | 6 |
| pj6031 | Respondent's partner has had schizophrenia since study child was 5 | Parent | no | mental health problems | 6yrs1m | 5 | 6 |
| p2028 | Mother attempted suicide since the study child's 6th birthday | Parent | yes | mental health problems | 9yrs2m | 6 | 7 |
| pm2028 | Father attempted suicide since the study child's 6th birthday | Parent | yes | mental health problems | 9yrs2m | 6 | 7 |
| pm1020 | Father has had schizophrenia in last 3 years | Parent | no | mental health problems | 9yrs2m | 6 | 9 |
| p1020 | Mother has had schizophrenia in last 3 years | Parent | no | mental health problems | 9yrs2m | 6 | 9 |
| p1054 | Frequency mother has taken pills for depression in last 2 years | Parent | no | mental health problems | 9yrs2m | 7 | 9 |
| p3031 | Husband/partner has had schizophrenia, in last 2 years | Parent | no | mental health problems | 9yrs2m | 7 | 9 |
| pm1054 | Frequency father has taken pills for depression in last 2 years | Parent | no | mental health problems | 9yrs2m | 7 | 9 |
| pm3031 | Wife/partner has had schizophrenia, in last 2 years | Parent | no | mental health problems | 9yrs2m | 7 | 9 |
| n1042 | Mother has ever had bulimia | Parent | no | mental health problems | 8yrs1m | 8 | 8 |
| n1059 | Mother has ever had schizophrenia | Parent | no | mental health problems | 8yrs1m | 8 | 8 |
| n1060 | Mother has ever had anorexia nervosa | Parent | no | mental health problems | 8yrs1m | 8 | 8 |
| pl1042 | Respondent has ever had bulimia | Parent | no | mental health problems | 8yrs1m | 8 | 8 |
| pl1059 | Respondent has ever had schizophrenia | Parent | no | mental health problems | 8yrs1m | 8 | 8 |
| pl1060 | Respondent has ever had anorexia nervosa | Parent | no | mental health problems | 8yrs1m | 8 | 8 |
| p2028_dup | Mother attempted suicide since the study child's 6th birthday | Parent | no | mental health problems | 9yrs2m | 8 | 9 |
| pm2028_dup | Father attempted suicide since the study child's 6th birthday | Parent | no | mental health problems | 9yrs2m | 8 | 9 |
| pn4100 | Respondent has taken medicines for depression in the past 12 months | Parent | no | mental health problems | 10yrs2m | 9 | 10 |
| pn4110 | Respondent has taken medicines for anxiety/nerves in the past 12 months | Parent | no | mental health problems | 10yrs2m | 9 | 10 |
| q4100 | Mother used medicine in last 12 months for depression | Parent | no | mental health problems | 10yrs2m | 9 | 10 |
| q4110 | Mother used medicine in last 12 months for anxiety/nerves | Parent | no | mental health problems | 10yrs2m | 9 | 10 |
| r5028 | Respondent attempted suicide since the study child's 9th birthday | Parent | yes | mental health problems | 11yrs2m | 9 | 10 |
| pp5028 | Respondent attempted suicide since the study child's 9th birthday | Parent | yes | mental health problems | 11yrs2m | 9 | 10 |
| pp5028_dup | Respondent attempted suicide since the study child's 9th birthday | Parent | no | mental health problems | 11yrs2m | 11 | 11 |
| r5028_dup | Respondent attempted suicide since the study child's 9th birthday | Parent | no | mental health problems | 11yrs2m | 11 | 11 |
| pq1020 | Partner has had schizophrenia in the last two years | Parent | no | mental health problems | 12yrs1m | 10 | 12 |
| pq3031 | Partner's partner had schizophrenia in the last 2 years | Parent | no | mental health problems | 12yrs1m | 10 | 12 |
| s1020 | Mother has had schizophrenia in last 2 years | Parent | no | mental health problems | 12yrs1m | 10 | 12 |
| s3031 | Mother's partner has had schizophrenia since study child's 10th birthday | Parent | no | mental health problems | 12yrs1m | 10 | 12 |
| pp2002 | Respondent has ever had bulimia | Parent | no | mental health problems | 11yrs2m | 11 | 11 |
| pp2019 | Respondent has ever had schizophrenia | Parent | no | mental health problems | 11yrs2m | 11 | 11 |
| pp2020 | Respondent has ever had anorexia nervosa | Parent | no | mental health problems | 11yrs2m | 11 | 11 |
| r2002 | Respondent has ever had bulimia | Parent | no | mental health problems | 11yrs2m | 11 | 11 |
| r2019 | Respondent has ever had schizophrenia | Parent | no | mental health problems | 11yrs2m | 11 | 11 |
| r2020 | Respondent has ever had anorexia nervosa | Parent | no | mental health problems | 11yrs2m | 11 | 11 |
| pq4100 | Partner has used medicine for depression in the last 12 months | Parent | no | mental health problems | 12yrs1m | 11 | 12 |
| pq4110 | Partner has used medicine for anxiety or nerves in the last 12 months | Parent | no | mental health problems | 12yrs1m | 11 | 12 |
| s4100 | Mother has taken medication for depression in the past 12 months | Parent | no | mental health problems | 12yrs1m | 11 | 12 |
| s4110 | Mother has taken medication for anxiety or nerves in the past 12 months | Parent | no | mental health problems | 12yrs1m | 11 | 12 |
| ccs6501 | YP's mum has hurt themselves on purpose | Child | no | mental health problems | 16yrs | 16 | 16 |
| ccs6502 | YP's dad has hurt themselves on purpose | Child | no | mental health problems | 16yrs | 16 | 16 |
| t5404 | Frequency respondent taken pills for depression in last two years | Parent | no | mental health problems | 2010 | 16.5 | 18.5 |
| t3327 | Respondent attempted suicide in last year | Parent | no | mental health problems | 2010 | 17.5 | 18.5 |
| t3255 | EPDS Total score | Parent | no | mental health problems | 2010 | 18.5 | 18.5 |
| t5300 | Respondent ever admitted to hospital for psychiatric or mental health problems | Parent | no | mental health problems | 2010 | 18.5 | 18.5 |
| t5305 | Study child's biological father ever admitted to hospital for psychiatric or mental health problems | Parent | no | mental health problems | 2010 | 18.5 | 18.5 |
| t5320 | Respondent has ever had an illness that included hearing voices or seeing things that were not there | Parent | no | mental health problems | 2010 | 18.5 | 18.5 |
| t5340 | Respondent ever had an illness with paranoid delusions or developed unusual false beliefs | Parent | no | mental health problems | 2010 | 18.5 | 18.5 |
| t5345 | Study child's biological father ever had an illness with paranoid delusions or developed unusual false beliefs | Parent | no | mental health problems | 2010 | 18.5 | 18.5 |
| t5360 | Respondent ever suffered from schizophrenia | Parent | no | mental health problems | 2010 | 18.5 | 18.5 |
| t5365 | Study child's biological father ever suffered from schizophrenia | Parent | no | mental health problems | 2010 | 18.5 | 18.5 |
| t5380 | Respondent ever suffered from a manic illness | Parent | no | mental health problems | 2010 | 18.5 | 18.5 |
| t5385 | Study child's biological father ever suffered from a manic illness | Parent | no | mental health problems | 2010 | 18.5 | 18.5 |
| fa5404 | Pills for depression | Parent | no | mental health problems | 2011-2013 | 18.5 | 20.5 |
| fa3327 | In last year, attempted suicide | Parent | no | mental health problems | 2011-2013 | 19.5 | 20.5 |
| a600 | Mums opinion of neighbourhood | Parent | no | neighbourhood | 8w gest | -1 | -1 |
| f900 | MUMS opinion of neighbourhood to live in | Parent | no | neighbourhood | 8m | 0.67 | 0.67 |
| g860 | Mums opinion of neighbourhood | Parent | no | neighbourhood | 1yrs9m | 2 | 2 |
| h778 | Mum feels neighbourhood is good place to live | Parent | no | neighbourhood | 2yrs9m | 3 | 3 |
| k7020 | Mothers opinion of her neighbourhood as place to live | Parent | no | neighbourhood | 5yrs1m | 5 | 5 |
| m2250 | Mother thinks neighbourhood is a good place | Parent | no | neighbourhood | 7yrs1m | 7 | 7 |
| q2240 | Mother's opinion of neighbourhood as a place to live | Parent | no | neighbourhood | 10yrs2m | 10 | 10 |
| ccr206 | Respondent on the whole is happy living in their neighbourhood | Child | no | neighbourhood | 14yrs | 14 | 14 |
| fh8092 | Degree to which YP feels their neighbourhood has a good reputation | Child | no | neighbourhood | 15.5yrs | 15.5 | 15.5 |
| fh8090 | Degree to which YP would rather live in another area than their own neighbourhood | Child | no | neighbourhood | 15.5yrs | 15.5 | 15.5 |
| fh8091 | Degree to which YP feels their neighbourhood has more crime than other areas | Child | no | neighbourhood | 15.5yrs | 15.5 | 15.5 |
| ccxd750 | YP believes the area in which they live is a place they enjoy living in | Child | no | neighbourhood | 17.5yrs | 17.5 | 17.5 |
| t2035 | Respondent's opinion of her neighbourhood as place to live | Parent | no | neighbourhood | 2010 | 18.5 | 18.5 |
| fa2035 | Opinion of neighbourhood as place to live | Parent | no | neighbourhood | 2011-2013 | 20.5 | 20.5 |
| ypc1810 | When growing up respondent felt loved | Child | yes | parent child bond | 23yrs | 0 | 16 |
| g634 | Partner seems very close to child | Parent | no | parent child bond | 1yrs9m | 2 | 2 |
| h514 | Mum feels partner is close to child | Parent | no | parent child bond | 2yrs9m | 3 | 3 |
| j567 | MUM Feels Close to CH | Parent | no | parent child bond | 3yrs11m | 4 | 4 |
| j577 | Partner Feels Close to CH | Parent | no | parent child bond | 3yrs11m | 4 | 4 |
| pg4167 | Partner feels very close to child | Parent | no | parent child bond | 3yrs11m | 4 | 4 |
| pg4177 | Partner's partner feels very close to child | Parent | no | parent child bond | 3yrs11m | 4 | 4 |
| l6054 | Frequency respondent's partner seems to feel very close to study child | Parent | no | parent child bond | 6yrs1m | 6 | 6 |
| pj6054 | Respondent's partner feels very close to study child | Parent | no | parent child bond | 6yrs1m | 6 | 6 |
| m3357 | Mother seems to feel close to study child | Parent | no | parent child bond | 7yrs1m | 7 | 7 |
| m3367 | Partner seems to feel close to study child | Parent | no | parent child bond | 7yrs1m | 7 | 7 |
| pk3357 | Partner feels very close to study child | Parent | no | parent child bond | 7yrs1m | 7 | 7 |
| pk3367 | Partner's partner seems to feel very close to study child | Parent | no | parent child bond | 7yrs1m | 7 | 7 |
| n8377 | Mother feels very close to study child | Parent | no | parent child bond | 8yrs1m | 8 | 8 |
| n8387 | Partner is very close to study child | Parent | no | parent child bond | 8yrs1m | 8 | 8 |
| p3054 | Mother feels father/partner seems to feel very close to the study child | Parent | no | parent child bond | 9yrs2m | 9 | 9 |
| pm3054 | Father feels wife/partner seems to feel very close to the study child | Parent | no | parent child bond | 9yrs2m | 9 | 9 |
| pq3054 | Partner's partner seems to feel very close to study child | Parent | no | parent child bond | 12yrs1m | 12 | 12 |
| s3054 | Mother's partner seems very close to study child | Parent | no | parent child bond | 12yrs1m | 12 | 12 |
| ff4530 | Adult/child interaction - harmony | Child | no | parent child bond | 12.5yrs | 12.5 | 12.5 |
| fjpc2000 | How close YP feels to their parents | Child | no | parent child bond | 17.5yrs | 17.5 | 17.5 |
| b598 | Convicted of an offence since PREG | Parent | no | parent convicted | 18w gest | -1 | -1 |
| pb188a | Convicted of offence since PTNR PREG,Y/N | Parent | no | parent convicted | 18w gest | -1 | -1 |
| e428 | Convicted since MID PREG | Parent | no | parent convicted | 8w | 0.16 | 0.16 |
| pc228a | Convicted since MID PREG, Y/N | Parent | no | parent convicted | 8w | 0.16 | 0.16 |
| f249a | Court conviction | Parent | no | parent convicted | 8m | 0.67 | 0.67 |
| pd249a | Convicted of Offence Since Baby Born | Parent | no | parent convicted | 8m | 0.67 | 0.67 |
| g329 | Mum convicted of offence >CH8MTHs | Parent | no | parent convicted | 1yrs9m | 0.67 | 2 |
| g329a | Mum convicted of offence >CH8MTHs | Parent | no | parent convicted | 1yrs9m | 0.67 | 2 |
| pe329a | Convicted of Offence Y/N | Parent | no | parent convicted | 1yrs9m | 0.67 | 2 |
| h239 | Whether mum was convicted of an offence since study child was 18 months old and effect this had | Parent | no | parent convicted | 2yrs9m | 1.5 | 3 |
| pf5029 | Partner was convicted of an offence since study child was 18 months old | Parent | no | parent convicted | 2yrs9m | 1.5 | 3 |
| j329 | MUM Convicted of Offence> CH 30 MTHs | Parent | no | parent convicted | 3yrs11m | 2.5 | 4 |
| pg3029 | Degree to which a criminal conviction affected partner since child was 2.5 years old | Parent | no | parent convicted | 3yrs11m | 2.5 | 4 |
| k4029 | Mother was convicted of an offence in past year | Parent | no | parent convicted | 5yrs1m | 4 | 5 |
| ph4029 | Respondent's assessment of how much being convicted of an offence in the last year has affected them | Parent | no | parent convicted | 5yrs1m | 4 | 5 |
| l4029 | Respondent convicted of an offence since study child's 5th birthday | Parent | no | parent convicted | 6yrs1m | 5 | 6 |
| pj4029 | Respondent's assessment of how much being convicted of an offence since study child's 5th birthday has affected them | Parent | no | parent convicted | 6yrs1m | 5 | 6 |
| p2029 | Mother was convicted of an offence since the study child's 6th birthday | Parent | yes | parent convicted | 9yrs2m | 6 | 7 |
| pm2029 | Father was convicted of an offence since the study child's 6th birthday | Parent | yes | parent convicted | 9yrs2m | 6 | 7 |
| p2029_dup | Mother was convicted of an offence since the study child's 6th birthday | Parent | no | parent convicted | 9yrs2m | 8 | 9 |
| pm2029_dup | Father was convicted of an offence since the study child's 6th birthday | Parent | no | parent convicted | 9yrs2m | 8 | 9 |
| pp5029 | Respondent has been convicted of an offence since the study child's 9th birthday | Parent | yes | parent convicted | 11yrs2m | 9 | 10 |
| r5029 | Respondent was convicted of an offence since study child's 9th birthday | Parent | yes | parent convicted | 11yrs2m | 9 | 10 |
| pp5029_dup | Respondent has been convicted of an offence since the study child's 9th birthday | Parent | no | parent convicted | 11yrs2m | 11 | 11 |
| r5029_dup | Respondent was convicted of an offence since study child's 9th birthday | Parent | no | parent convicted | 11yrs2m | 11 | 11 |
| pq5014 | Partner has been convicted of an offence other then speeding in the last year | Parent | no | parent convicted | 12yrs1m | 11 | 12 |
| s5014 | Mother was convicted of an offence in the last year | Parent | no | parent convicted | 12yrs1m | 11 | 12 |
| t3328 | Respondent was convicted of an offence in last year | Parent | no | parent convicted | 2010 | 17.5 | 18.5 |
| fa3328 | In last year, was convicted of an offence | Parent | no | parent convicted | 2011-2013 | 19.5 | 20.5 |
| b578 | Divorced since PREG | Parent | no | parental separation | 18w gest | -1 | -1 |
| b587 | Separated since PREG | Parent | no | parental separation | 18w gest | -1 | -1 |
| pb168 | Divorced since PTNR PREG | Parent | no | parental separation | 18w gest | -1 | -1 |
| pb177 | Separated since PTNR PREG | Parent | no | parental separation | 18w gest | -1 | -1 |
| f228 | Divorce >CH born | Parent | no | parental separation | 8m | 0 | 0.67 |
| f237 | Separation from PTNR >CH born | Parent | no | parental separation | 8m | 0 | 0.67 |
| pd228 | Divorced Since Baby Born | Parent | no | parental separation | 8m | 0 | 0.67 |
| pd237 | Separated Since Baby Born | Parent | no | parental separation | 8m | 0 | 0.67 |
| e408 | Divorced since MID PREG | Parent | no | parental separation | 8w | -1 | 0.16 |
| e417 | Separated since MID PREG | Parent | no | parental separation | 8w | -1 | 0.16 |
| pc208 | Divorced since MID PREG | Parent | no | parental separation | 8w | -1 | 0.16 |
| pc217 | You & PTNR separated since MID PREG | Parent | no | parental separation | 8w | -1 | 0.16 |
| g308 | Mum divorced >CH8MTHs | Parent | no | parental separation | 1yrs9m | 0.67 | 2 |
| g317 | Mum and partner separated >CH8MTHs | Parent | no | parental separation | 1yrs9m | 0.67 | 2 |
| pe308 | Divorced | Parent | no | parental separation | 1yrs9m | 0.67 | 2 |
| pe317 | Separated From Partner | Parent | no | parental separation | 1yrs9m | 0.67 | 2 |
| h218 | Whether mum got divorced since study child was 18 months old and effect this had | Parent | no | parental separation | 2yrs9m | 1.5 | 3 |
| h227 | Whether mum and partner separated since study child was 18 months old and effect this had | Parent | no | parental separation | 2yrs9m | 1.5 | 3 |
| pf5008 | Partner was divorced since study child was 18 months old | Parent | no | parental separation | 2yrs9m | 1.5 | 3 |
| pf5017 | Partner and partner's partner have separated since study child was 18 months old | Parent | no | parental separation | 2yrs9m | 1.5 | 3 |
| j308 | MUM Divorced> CH 30 MTHs | Parent | no | parental separation | 3yrs11m | 2.5 | 4 |
| j317 | MUM & PTR Separated> CH 30 MTHs | Parent | no | parental separation | 3yrs11m | 2.5 | 4 |
| pg3008 | Degree to which divorce affected partner since child was 2.5 years old | Parent | no | parental separation | 3yrs11m | 2.5 | 4 |
| pg3017 | Degree to which separation affected partner since child was 2.5 years old | Parent | no | parental separation | 3yrs11m | 2.5 | 4 |
| k4008 | Mother was divorced in past year | Parent | no | parental separation | 5yrs1m | 4 | 5 |
| k4017 | Mother and partner separated in past year | Parent | no | parental separation | 5yrs1m | 4 | 5 |
| ph4017 | Respondent's assessment of how much separating from their partner in the last year has affected them | Parent | no | parental separation | 5yrs1m | 4 | 5 |
| l4008 | Respondent was divorced since study child's 5th birthday | Parent | no | parental separation | 6yrs1m | 5 | 6 |
| l4017 | Respondent separated from partner since study child's 5th birthday | Parent | no | parental separation | 6yrs1m | 5 | 6 |
| pj4008 | Respondent's assessment of how much divorce since study child's 5th birthday has affected them | Parent | no | parental separation | 6yrs1m | 5 | 6 |
| pj4017 | Respondent's assessment of how much separating from partner since study child's 5th birthday has affected them | Parent | no | parental separation | 6yrs1m | 5 | 6 |
| p2008 | Mother was divorced since the study child's 6th birthday | Parent | yes | parental separation | 9yrs2m | 6 | 7 |
| p2017 | Mother and husband/partner separated since the study child's 6th birthday | Parent | yes | parental separation | 9yrs2m | 6 | 7 |
| pm2008 | Father was divorced since the study child's 6th birthday | Parent | yes | parental separation | 9yrs2m | 6 | 7 |
| pm2017 | Father and wife/partner separated since the study child's 6th birthday | Parent | yes | parental separation | 9yrs2m | 6 | 7 |
| p3003 | Same partner/husband as mother had when study child had 6th birthday | Parent | no | parental separation | 9yrs2m | 6 | 9 |
| pm3003 | Same partner/wife as father had when study child had 6th birthday | Parent | no | parental separation | 9yrs2m | 6 | 9 |
| p2008_dup | Mother was divorced since the study child's 6th birthday | Parent | no | parental separation | 9yrs2m | 8 | 9 |
| p2017_dup | Mother and husband/partner separated since the study child's 6th birthday | Parent | no | parental separation | 9yrs2m | 8 | 9 |
| pm2008_dup | Father was divorced since the study child's 6th birthday | Parent | no | parental separation | 9yrs2m | 8 | 9 |
| pm2017_dup | Father and wife/partner separated since the study child's 6th birthday | Parent | no | parental separation | 9yrs2m | 8 | 9 |
| pp5008 | Respondent has divorced since the study child's 9th birthday | Parent | yes | parental separation | 11yrs2m | 9 | 10 |
| pp5015 | Respondent's wife/partner went away since the study child's 9th birthday | Parent | yes | parental separation | 11yrs2m | 9 | 10 |
| pp5017 | Respondent has separated from wife/partner since the study child's 9th birthday | Parent | yes | parental separation | 11yrs2m | 9 | 10 |
| r5008 | Respondent has been divorced since child's 9th birthday | Parent | yes | parental separation | 11yrs2m | 9 | 10 |
| r5017 | Respondent separated from husband/partner since the study child's 9th birthday | Parent | yes | parental separation | 11yrs2m | 9 | 10 |
| pq3003 | Partner's current partner is the same as on the study child's 9th birthday | Parent | no | parental separation | 12yrs1m | 9 | 12 |
| s3003 | Mother's current partner is the same as on study child's 9th birthday | Parent | no | parental separation | 12yrs1m | 9 | 12 |
| pp5008_dup | Respondent has divorced since the study child's 9th birthday | Parent | no | parental separation | 11yrs2m | 11 | 11 |
| pp5015_dup | Respondent's wife/partner went away since the study child's 9th birthday | Parent | no | parental separation | 11yrs2m | 11 | 11 |
| pp5017_dup | Respondent has separated from wife/partner since the study child's 9th birthday | Parent | no | parental separation | 11yrs2m | 11 | 11 |
| r5008_dup | Respondent has been divorced since child's 9th birthday | Parent | no | parental separation | 11yrs2m | 11 | 11 |
| r5017_dup | Respondent separated from husband/partner since the study child's 9th birthday | Parent | no | parental separation | 11yrs2m | 11 | 11 |
| ccs2050 | YP's parents have divorced/separated since the age of 12 | Child | no | parental separation | 16yrs | 12 | 16 |
| fjle112 | In the last year YP's parents have divorced | Child | no | parental separation | 17.5yrs | 16.5 | 17.5 |
| fjle114 | In the last year YP's parents have separated | Child | no | parental separation | 17.5yrs | 16.5 | 17.5 |
| t3308 | Respondent was divorced in last year | Parent | no | parental separation | 2010 | 17.5 | 18.5 |
| t3316 | Respondent and partner separated in last year | Parent | no | parental separation | 2010 | 17.5 | 18.5 |
| fa3308 | In last year, got divorced | Parent | no | parental separation | 2011-2013 | 19.5 | 20.5 |
| fa3316 | In last year, separated from partner | Parent | no | parental separation | 2011-2013 | 19.5 | 20.5 |
| f246 | PTNR physically cruel to CHDR >CH born | Parent | no | physical abuse | 8m | 0 | 0.67 |
| f247 | MUM physically cruel to CHDR >CH born | Parent | no | physical abuse | 8m | 0 | 0.67 |
| ypb8002 | Frequency adult in family pushed, grabbed or shoved respondent before age of 11 | Child | yes | physical abuse | 22yrs | 0 | 11 |
| ypb8003 | Frequency adult in family smacked respondent for discipline before age of 11 | Child | yes | physical abuse | 22yrs | 0 | 11 |
| ypb8006 | Frequency adult in family actually kicked, punched, hit respondent with something that could hurt respondent or physically attacked respondent in another way before age of 11 | Child | yes | physical abuse | 22yrs | 0 | 11 |
| ypb8007 | Frequency adult in family hit respondent so hard it left bruises or marks before age of 11 | Child | yes | physical abuse | 22yrs | 0 | 11 |
| ypc1811 | When growing up people in respondent's family hit them so hard that it left them with bruises or marks | Child | yes | physical abuse | 23yrs | 0 | 16 |
| pc226 | PTNR physical-cruel to CH since MID PREG | Parent | no | physical abuse | 8w | -1 | 0.16 |
| pd246 | Ptnr Physically Cruel To Children | Parent | no | physical abuse | 8m | 0.67 | 0.67 |
| pd247 | Self Physically Cruel To Children | Parent | no | physical abuse | 8m | 0.67 | 0.67 |
| g326 | Partner physically cruel to children >CH8MTHs | Parent | no | physical abuse | 1yrs9m | 0.67 | 2 |
| g327 | Mum physically cruel to children >CH8MTHs | Parent | no | physical abuse | 1yrs9m | 0.67 | 2 |
| pe326 | Partner Physically Cruel to Child | Parent | no | physical abuse | 1yrs9m | 0.67 | 2 |
| pe327 | Self Physically Cruel to Child | Parent | no | physical abuse | 1yrs9m | 0.67 | 2 |
| h236 | Whether partner was physically cruel to children since study child was 18 months old and effect this had | Parent | no | physical abuse | 2yrs9m | 1.5 | 3 |
| h237 | Whether mum was physically cruel to children since study child was 18 months old and effect this had | Parent | no | physical abuse | 2yrs9m | 1.5 | 3 |
| pf5026 | Partner's partner was physically cruel to their children since study child was 18 months old | Parent | no | physical abuse | 2yrs9m | 1.5 | 3 |
| pf5027 | Partner was physically cruel to their children since study child was 18 months old | Parent | no | physical abuse | 2yrs9m | 1.5 | 3 |
| j326 | PTR PHYS Cruel to CDRN> CH 30 MTHs | Parent | no | physical abuse | 3yrs11m | 2.5 | 4 |
| j327 | MUM PHYS Cruel to CDRN> CH 30 MTHs | Parent | no | physical abuse | 3yrs11m | 2.5 | 4 |
| pg3026 | Degree to which physical cruelty from a partner to children affected partner since study child was 2.5 years old | Parent | no | physical abuse | 3yrs11m | 2.5 | 4 |
| pg3027 | Degree to which partner being physically cruel to children affected partner since study child was 2.5 years old | Parent | no | physical abuse | 3yrs11m | 2.5 | 4 |
| k4026 | Mothers partner was physically cruel to children in past year | Parent | no | physical abuse | 5yrs1m | 4 | 5 |
| k4027 | Mother was physically cruel to children in past year | Parent | no | physical abuse | 5yrs1m | 4 | 5 |
| ph4026 | Respondent's assessment how much their partner being physically cruel to the children in the last year has affected them | Parent | no | physical abuse | 5yrs1m | 4 | 5 |
| ph4027 | Respondent's assessment of how much being physically cruel to the children in the last year has affected them | Parent | no | physical abuse | 5yrs1m | 4 | 5 |
| l4026 | Respondent's partner physically cruel to respondent's children since study child's 5th birthday | Parent | no | physical abuse | 6yrs1m | 5 | 6 |
| l4027 | Respondent physically cruel to own children since study child's 5th birthday | Parent | no | physical abuse | 6yrs1m | 5 | 6 |
| pj4026 | Respondent's assessment of how much partner's physical cruelty to children since study child's 5th birthday has affected them | Parent | no | physical abuse | 6yrs1m | 5 | 6 |
| pj4027 | Respondent's assessment of how much being physically cruel to children since study child's 5th birthday has affected them | Parent | no | physical abuse | 6yrs1m | 5 | 6 |
| pm2026 | Father's wife/partner was physically cruel to his children since the study child's 6th birthday | Parent | yes | physical abuse | 9yrs2m | 6 | 7 |
| pm2027 | Father was physically cruel to his children since the study child's 6th birthday | Parent | yes | physical abuse | 9yrs2m | 6 | 7 |
| p2026 | Mother's husband/partner was physically cruel to her children since the study child's 6th birthday | Parent | yes | physical abuse | 9yrs2m | 6 | 7 |
| p2027 | Mother was physically cruel to her children since the study child's 6th birthday | Parent | yes | physical abuse | 9yrs2m | 6 | 7 |
| p2026_dup | Mother's husband/partner was physically cruel to her children since the study child's 6th birthday | Parent | no | physical abuse | 9yrs2m | 8 | 9 |
| p2027_dup | Mother was physically cruel to her children since the study child's 6th birthday | Parent | no | physical abuse | 9yrs2m | 8 | 9 |
| pm2026_dup | Father's wife/partner was physically cruel to his children since the study child's 6th birthday | Parent | no | physical abuse | 9yrs2m | 8 | 9 |
| pm2027_dup | Father was physically cruel to his children since the study child's 6th birthday | Parent | no | physical abuse | 9yrs2m | 8 | 9 |
| pp5026 | Respondent's wife/partner was physically cruel to their children since the study child's 9th birthday | Parent | yes | physical abuse | 11yrs2m | 9 | 10 |
| pp5027 | Respondent was physically cruel to their children since the study child's 9th birthday | Parent | yes | physical abuse | 11yrs2m | 9 | 10 |
| r5026 | Respondent's husband/partner was physically cruel to their children since study child's 9th birthday | Parent | yes | physical abuse | 11yrs2m | 9 | 10 |
| r5027 | Respondent was physically cruel to their children since the study child's 9th birthday | Parent | yes | physical abuse | 11yrs2m | 9 | 10 |
| pp5026_dup | Respondent's wife/partner was physically cruel to their children since the study child's 9th birthday | Parent | no | physical abuse | 11yrs2m | 11 | 11 |
| pp5027_dup | Respondent was physically cruel to their children since the study child's 9th birthday | Parent | no | physical abuse | 11yrs2m | 11 | 11 |
| r5026_dup | Respondent's husband/partner was physically cruel to their children since study child's 9th birthday | Parent | no | physical abuse | 11yrs2m | 11 | 11 |
| r5027_dup | Respondent was physically cruel to their children since the study child's 9th birthday | Parent | no | physical abuse | 11yrs2m | 11 | 11 |
| ypb8052 | Frequency adult in family pushed, grabbed or shoved respondent between ages of 11 and 17 | Child | yes | physical abuse | 22yrs | 11 | 17 |
| ypb8053 | Frequency adult in family smacked respondent for discipline between ages of 11 and 17 | Child | yes | physical abuse | 22yrs | 11 | 17 |
| ypb8056 | Frequency adult in family actually kicked, punched, hit respondent with something that could hurt respondent or physically attacked respondent in another way between ages of 11 and 17 | Child | yes | physical abuse | 22yrs | 11 | 17 |
| ypb8057 | Frequency adult in family hit respondent so hard it left bruises or marks between ages of 11 and 17 | Child | yes | physical abuse | 22yrs | 11 | 17 |
| t3325 | Respondent's partner was physically cruel to respondent's children in last year | Parent | no | physical abuse | 2010 | 17.5 | 18.5 |
| t3326 | Respondent was physically cruel to own children in last year | Parent | no | physical abuse | 2010 | 17.5 | 18.5 |
| fa3325 | In last year, partner was physically cruel to respondent's children | Parent | no | physical abuse | 2011-2013 | 19.5 | 20.5 |
| fa3326 | In last year, respondent was physically cruel to respondent's children | Parent | no | physical abuse | 2011-2013 | 19.5 | 20.5 |
| kb067 | NO of HOSP admissons | Parent | no | physical ill child | 6m | 0.5 | 0.5 |
| kd058 | NO of Times Child Hospitalized | Parent | no | physical ill child | 18m | 1.5 | 1.5 |
| kj061 | Times CH Admitted to HOSP Past 12 Months | Parent | no | physical ill child | 42m | 2.5 | 3.5 |
| kl046 | Number of times child admitted to hospital since age 3 | Parent | no | physical ill child | 57m | 3 | 5 |
| kn1051 | Number of times Child was admitted to hospital in past 15 months | Parent | no | physical ill child | 69m | 4.5 | 6 |
| kq041 | No. Times CH Admitted To Hosp Past Year | Parent | no | physical ill child | 81m | 6 | 7 |
| ks1071 | Number of times child admitted to hospital in past 2 years | Parent | no | physical ill child | 105m | 7 | 9 |
| sa038 | Child has medical conditions | School | no | physical ill child | school year 3 | 7.5 | 7.5 |
| ta1061 | No. times teenager admitted to hospital since 9th birthday | Parent | no | physical ill child | 13yrs | 9 | 13 |
| se037 | Child has physical disabilities | School | no | physical ill child | school year 6 | 10.5 | 10.5 |
| se038 | Child has medical conditions | School | no | physical ill child | school year 6 | 10.5 | 10.5 |
| pd012 | Times Stayed in Hosp Since Baby Born | Parent | no | physical ill parent | 8m | 0 | 0.67 |
| pe101 | Times Stayed in Hospital CH > 8 Months | Parent | no | physical ill parent | 1yrs9m | 0.67 | 2 |
| h061 | No of times Mum has been hospitalised since study child was 18 months old | Parent | no | physical ill parent | 2yrs9m | 1.5 | 3 |
| pf1111 | Number of times partner has had to go and stay in hospital since the study child was 18 months old | Parent | no | physical ill parent | 2yrs9m | 1.5 | 3 |
| g081 | No. of times mum in hospital | Parent | no | physical ill parent | 1yrs9m | 2 | 2 |
| pg1111 | Number of times partner has been admitted to hospital since study child was 3 years old | Parent | no | physical ill parent | 3yrs11m | 3 | 4 |
| j071 | NO of Times Mum Hospitalized | Parent | no | physical ill parent | 3yrs11m | 4 | 4 |
| k1131 | Number of times mother admitted to hospital since child was 4 | Parent | no | physical ill parent | 5yrs1m | 4 | 5 |
| ph1131 | Number of times respondent has stayed in hospital since study child was 4 years old | Parent | no | physical ill parent | 5yrs1m | 4 | 5 |
| l3181 | Number of times respondent admitted to hospital since study child was 5 | Parent | no | physical ill parent | 6yrs1m | 5 | 6 |
| pj3181 | Number of times respondent has been admitted to hospital since study child was 5 | Parent | no | physical ill parent | 6yrs1m | 5 | 6 |
| l3035 | Respondent has had/continued to have cancer since study child's 5th birthday | Parent | no | physical ill parent | 6yrs1m | 5 | 6 |
| pj3035 | Respondent has suffered from cancer since child's 5th birthday | Parent | no | physical ill parent | 6yrs1m | 5 | 6 |
| p1171 | Number of times mother admitted to hospital since study child's 6th birthday | Parent | no | physical ill parent | 9yrs2m | 6 | 9 |
| pm1171 | Number of times father admitted to hospital since study child's 6th birthday | Parent | no | physical ill parent | 9yrs2m | 6 | 9 |
| p1035 | Mother has had cancer in last 3 years | Parent | no | physical ill parent | 9yrs2m | 6 | 9 |
| pm1035 | Father has had cancer in last 3 years | Parent | no | physical ill parent | 9yrs2m | 6 | 9 |
| pp2101 | Number of times respondent has been admitted to hospital since study child's 9th birthday | Parent | no | physical ill parent | 11yrs2m | 9 | 11 |
| r2101 | Number of times respondent admitted to hospital since child's 9th birthday | Parent | no | physical ill parent | 11yrs2m | 9 | 11 |
| pq1171 | Number of times partner was admitted to hospital since study child's 9th birthday | Parent | no | physical ill parent | 12yrs1m | 9 | 12 |
| s1171 | Number of times mother has been admitted to hospital since study child's 9th birthday | Parent | no | physical ill parent | 12yrs1m | 9 | 12 |
| pq1035 | Partner has had cancer in the last two years | Parent | no | physical ill parent | 12yrs1m | 10 | 12 |
| s1035 | Mother has had cancer in last 2 years | Parent | no | physical ill parent | 12yrs1m | 10 | 12 |
| ypb8030 | Respondent was touched in a sexual way by adult or older child, or was forced to touch adult or older child in a sexual way, before age of 11 | Child | yes | sexual abuse | 22yrs | 0 | 11 |
| ypb8040 | Adult or older child forced, or attempted to force, respondent into any sexual activity by threatening or holding respondent down or hurting respondent in some way, before age of 11 | Child | yes | sexual abuse | 22yrs | 0 | 11 |
| ypc1813 | When growing up someone molested respondent (sexually) | Child | yes | sexual abuse | 23yrs | 0 | 16 |
| kd505a | CH Sexually Abused > 6 MTHS | Parent | no | sexual abuse | 18m | 0.5 | 1.5 |
| kf455a | Child sexually abused > 18 months, Y/N | Parent | no | sexual abuse | 30m | 1.5 | 2.5 |
| kj465 | CH was Sexually Abused Past 12 MTHs | Parent | no | sexual abuse | 42m | 2.5 | 3.5 |
| kl475 | Child was sexually abused since age 3 | Parent | no | sexual abuse | 57m | 3 | 5 |
| kn4005 | Child sexually abused in past 15 months | Parent | no | sexual abuse | 69m | 4.5 | 6 |
| kq365 | Child was sexually abused since his/her 5th birthday | Parent | no | sexual abuse | 81m | 5 | 7 |
| kt5005 | Since 7th birthday child has been sexually abused | Parent | no | sexual abuse | 105m | 7 | 9 |
| ypb8080 | Respondent was touched in a sexual way by adult or older child, or was forced to touch adult or older child in a sexual way, between ages of 11 and 17 | Child | yes | sexual abuse | 22yrs | 11 | 17 |
| ypb8090 | Adult or older child forced, or attempted to force, respondent into any sexual activity by threatening or holding respondent down or hurting respondent in some way, between ages of 11 and 17 | Child | yes | sexual abuse | 22yrs | 11 | 17 |
| sc household 12wgest | social class based on occupation partner | Parent | no | social class | 12w gest | -1 | -1 |
| sc household 18wgest | social class based on highest occupational level mother and partner | Parent | no | social class | 18w gest | -1 | -1 |
| sc household 32wgest | social class based on highest occupational level mother and partner | Parent | no | social class | 32w gest | 0 | 0 |
| sc household 8m | social class based on highest occupational level mother and partner | Parent | no | social class | 8m | 0.67 | 0.67 |
| sc household 2yr | social class based on highest occupational level mother and partner | Parent | no | social class | 1yrs9m | 2 | 2 |
| sc household 3yr | social class based on highest occupational level mother and partner | Parent | no | social class | 2yrs9m | 3 | 3 |
| sc household 4yr | social class based on highest occupational level mother and partner | Parent | no | social class | 3yrs11m | 4 | 4 |
| sc household 8yr | social class based on occupation partner | Parent | no | social class | 8yrs1m | 8 | 8 |
| f8fs110 | Happy with no. of friends | Child | no | social support child | 8.5yrs | 8.5 | 8.5 |
| f8fs112 | Friends understand Ch | Child | no | social support child | 8.5yrs | 8.5 | 8.5 |
| fdfs110 | Happy with no. of friends | Child | no | social support child | 10+yrs | 10 | 10 |
| fdfs117 | Friends understand child | Child | no | social support child | 10+yrs | 10 | 10 |
| fdfs111 | No. of close friends child has | Child | no | social support child | 10+yrs | 10 | 10 |
| fefs010 | No. close friends child has | Child | no | social support child | 11+yrs | 11 | 11 |
| ff5401 | Teenager is happy with number of friends | Child | no | social support child | 12.5yrs | 12.5 | 12.5 |
| ff5409 | Teenager believes friends understand them | Child | no | social support child | 12.5yrs | 12.5 | 12.5 |
| ff5402 | Number of close friends teenager has | Child | no | social support child | 12.5yrs | 12.5 | 12.5 |
| fg4120 | Teenager is happy with number of friends | Child | no | social support child | 13.5yrs | 13.5 | 13.5 |
| fg4128 | Teenager believes friends understand them | Child | no | social support child | 13.5yrs | 13.5 | 13.5 |
| fh8325 | Number of friends YP has altogether | Child | no | social support child | 15.5yrs | 15.5 | 15.5 |
| fh8331 | Frequency YPs friends support YP when they need them | Child | no | social support child | 15.5yrs | 15.5 | 15.5 |
| fjpc100 | YP is happy with the number of friends they have | Child | no | social support child | 17.5yrs | 17.5 | 17.5 |
| fjpc150 | YP talks to any of their friends about their problems | Child | no | social support child | 17.5yrs | 17.5 | 17.5 |
| fjpc200 | YP thinks that their friends understand them | Child | no | social support child | 17.5yrs | 17.5 | 17.5 |
| fjpc050 | Number of close friends YP has | Child | no | social support child | 17.5yrs | 17.5 | 17.5 |
| d790 | No one to share feelings with | Parent | no | social support parent | 15w gest | -1 | -1 |
| pb130 | No one to share feelings with | Parent | no | social support parent | 18w gest | -1 | -1 |
| e600 | No-one to share feelings with | Parent | no | social support parent | 8w | 0.16 | 0.16 |
| pc330 | Have no-one to share feelings with | Parent | no | social support parent | 8w | 0.16 | 0.16 |
| f910 | No-one to share feelings with | Parent | no | social support parent | 8m | 0.67 | 0.67 |
| pd740 | Have No one To Share Feelings With | Parent | no | social support parent | 8m | 0.67 | 0.67 |
| g216 | Mum feels nobody to share feelings with | Parent | no | social support parent | 1yrs9m | 2 | 2 |
| pe200 | Have No-one to Share Feelings With | Parent | no | social support parent | 1yrs9m | 2 | 2 |
| k8020 | Mother feels she has no-one to share feelings with | Parent | no | social support parent | 5yrs1m | 5 | 5 |
| ph8020 | Respondent feels they have no one to share their feelings with | Parent | no | social support parent | 5yrs1m | 5 | 5 |
| l7020 | Degree to which respondent has no one to share their feelings with | Parent | no | social support parent | 6yrs1m | 6 | 6 |
| pj7020 | Respondent has no one to share their feelings with | Parent | no | social support parent | 6yrs1m | 6 | 6 |
| p4020 | Mother has no one to share her feelings with | Parent | no | social support parent | 9yrs2m | 9 | 9 |
| pm4020 | Father has no one to share his feelings with | Parent | no | social support parent | 9yrs2m | 9 | 9 |
| pq6020 | Partner has no one to share feelings with | Parent | no | social support parent | 12yrs1m | 12 | 12 |
| s6020 | Mother has no one to share feelings with | Parent | no | social support parent | 12yrs1m | 12 | 12 |
| b701 | Smoked cannabis in 1-3MTHS of PREG | Parent | yes | substance household | 18w gest | -1 | -1 |
| b702 | Smoked cannabis >3MTHS PREG | Parent | no | substance household | 18w gest | -1 | -1 |
| b714 | Hard drugs | Parent | no | substance household | 18w gest | -1 | -1 |
| d167 | Had drug addiction | Parent | no | substance household | 15w gest | -1 | -1 |
| d168 | Had alcoholism | Parent | no | substance household | 15w gest | -1 | -1 |
| pa187 | Had drug addiction | Parent | no | substance household | 12w gest | -1 | -1 |
| pa188 | Had alcoholism | Parent | no | substance household | 12w gest | -1 | -1 |
| pb098 | Hard drugs | Parent | no | substance household | 18w gest | -1 | -1 |
| e190 | FREQ of ganja use in last 2MTHS of PREG | Parent | yes | substance household | 8w | -1 | 0 |
| e203 | Hard drug in last 2 months | Parent | no | substance household | 8w | -1 | 0 |
| e192 | FREQ of ganja use since birth | Parent | no | substance household | 8w | 0 | 0.16 |
| e213 | Hard drug use since delivery | Parent | no | substance household | 8w | 0 | 0.16 |
| pc266 | FREQ cannabis smoked since birth | Parent | no | substance household | 8w | 0 | 0.16 |
| f061 | Cannabis use since CH born | Parent | no | substance household | 8m | 0 | 0.67 |
| f067 | Amphetamine use since CH born | Parent | no | substance household | 8m | 0 | 0.67 |
| f069 | Opiate or cocaine use since CH born | Parent | no | substance household | 8m | 0 | 0.67 |
| f527 | PTNR had alcoholism >CH born | Parent | no | substance household | 8m | 0 | 0.67 |
| pd061 | Used Cannabis/Marijuana Since Baby Born | Parent | no | substance household | 8m | 0 | 0.67 |
| pd066 | Used Amphetamines Since Baby Born | Parent | no | substance household | 8m | 0 | 0.67 |
| pd067 | Used Heroin, Cocaine Since Baby Born | Parent | no | substance household | 8m | 0 | 0.67 |
| pc276 | Hard drugs | Parent | no | substance household | 8w | 0.16 | 0.16 |
| g047 | Mum had cannabis >CH8MTHs | Parent | no | substance household | 1yrs9m | 0.67 | 2 |
| g053 | Mum had amphetamines >CH8MTHs | Parent | no | substance household | 1yrs9m | 0.67 | 2 |
| g056 | Mum had heroin meth coc >CH8MTHs | Parent | no | substance household | 1yrs9m | 0.67 | 2 |
| g613 | Partner alcoholic >CH8MTHs | Parent | no | substance household | 1yrs9m | 0.67 | 2 |
| pe062 | Taken Cannabis Since CH > 8 Months | Parent | no | substance household | 1yrs9m | 0.67 | 2 |
| pe067 | Taken Amphetamines Since CH > 8 Months | Parent | no | substance household | 1yrs9m | 0.67 | 2 |
| pe069 | Taken Heroin/Cocaine CH > 8 Months | Parent | no | substance household | 1yrs9m | 0.67 | 2 |
| h037 | Frequency Mum has taken cannabis since study child was 18 months old | Parent | no | substance household | 2yrs9m | 1.5 | 3 |
| h043 | Frequency Mum has taken amphetamines since study child was 18 months old | Parent | no | substance household | 2yrs9m | 1.5 | 3 |
| h046 | Frequency Mum has taken herion, methadone, crack or cocaine since study child was 18 months old | Parent | no | substance household | 2yrs9m | 1.5 | 3 |
| h498 | Partner had an alcohol problem since study child was 18 months old | Parent | no | substance household | 2yrs9m | 1.5 | 3 |
| pf1032 | Frequency partner has taken cannabis/marihuana since the study child was 18 months old | Parent | no | substance household | 2yrs9m | 1.5 | 3 |
| pf1037 | Frequency partner has taken amphetamines or other stimulants since the study child was 18 months old | Parent | no | substance household | 2yrs9m | 1.5 | 3 |
| pf1039 | Frequency partner has taken heroin/methadone/crack/cocaine since the study child was 18 months old | Parent | no | substance household | 2yrs9m | 1.5 | 3 |
| pg1032 | Frequency partner has used cannabis/marihuana in the past year | Parent | no | substance household | 3yrs11m | 3 | 4 |
| j042 | MUM took Cannabis >1 YR | Parent | no | substance household | 3yrs11m | 3 | 4 |
| j048 | MUM took Amphetemines >1 YR | Parent | no | substance household | 3yrs11m | 3 | 4 |
| j051 | MUM took Heroin >1 YR | Parent | no | substance household | 3yrs11m | 3 | 4 |
| j616 | Partner had Alcohol Problem> 1 YR | Parent | no | substance household | 3yrs11m | 3 | 4 |
| pg1037 | Frequency partner has taken amphetamines or other stimulants in the past year | Parent | no | substance household | 3yrs11m | 3 | 4 |
| pg1039 | Frequency partner has taken heroin, methadone, crack or cocaine in the past year | Parent | no | substance household | 3yrs11m | 3 | 4 |
| k1022 | Mother had alcohol problem in past year | Parent | no | substance household | 5yrs1m | 4 | 5 |
| k1042 | Frequency mother had cannabis/marijuana in past year | Parent | no | substance household | 5yrs1m | 4 | 5 |
| k1050 | Frequency mother had amphetamines in past year | Parent | no | substance household | 5yrs1m | 4 | 5 |
| k1053 | Frequency mother had heroin/methadone/cocaine in past year | Parent | no | substance household | 5yrs1m | 4 | 5 |
| ph1022 | Respondent had alcohol problems in the past year | Parent | no | substance household | 5yrs1m | 4 | 5 |
| ph1042 | Frequency in the past year respondent has taken cannabis/marihuana | Parent | no | substance household | 5yrs1m | 4 | 5 |
| ph1050 | Frequency in the past year respondent has taken amphetamines/other stimulants | Parent | no | substance household | 5yrs1m | 4 | 5 |
| ph1053 | Frequency in the past year respondent has taken heroin/methadone/crack/cocaine | Parent | no | substance household | 5yrs1m | 4 | 5 |
| pj3053 | Respondent has taken heroin, methadone, crack or cocaine since child's 5th birthday | Parent | no | substance household | 6yrs1m | 5 | 6 |
| l3022 | Respondent has had/continued to have alcohol problem since study child's 5th birthday | Parent | no | substance household | 6yrs1m | 5 | 6 |
| l3042 | Frequency respondent has taken cannabis/marihuana since study child's 5th birthday | Parent | no | substance household | 6yrs1m | 5 | 6 |
| l3050 | Frequency respondent has taken amphetamines/other stimulants since study child's 5th birthday | Parent | no | substance household | 6yrs1m | 5 | 6 |
| l3053 | Frequency respondent has taken heroin, methadone, crack, cocaine since study child's 5th birthday | Parent | no | substance household | 6yrs1m | 5 | 6 |
| l6032 | Respondent's partner has had a drink (alcohol) problem since study child was 5 years old | Parent | no | substance household | 6yrs1m | 5 | 6 |
| pj3022 | Respondent has suffered from alcohol problems since child's 5th birthday | Parent | no | substance household | 6yrs1m | 5 | 6 |
| pj3042 | Respondent has taken cannabis or marihuana since child's 5th birthday | Parent | no | substance household | 6yrs1m | 5 | 6 |
| pj3050 | Respondent has taken amphetamines or other stimulants since child's 5th birthday | Parent | no | substance household | 6yrs1m | 5 | 6 |
| pj6032 | Respondent's partner has had alcohol problems since study child was 5 | Parent | no | substance household | 6yrs1m | 5 | 6 |
| p1022 | Mother has had alcohol problem in last 3 years | Parent | no | substance household | 9yrs2m | 6 | 9 |
| pm1022 | Father has had alcohol problem in last 3 years | Parent | no | substance household | 9yrs2m | 6 | 9 |
| p1052 | Frequency mother has taken cannabis/marihuana in last 2 years | Parent | no | substance household | 9yrs2m | 7 | 9 |
| p1060 | Frequency mother has taken amphetamines or other stimulants in last 2 years | Parent | no | substance household | 9yrs2m | 7 | 9 |
| p1063 | Frequency mother has taken heroin, methadone, crack, cocaine in last 2 years | Parent | no | substance household | 9yrs2m | 7 | 9 |
| p3032 | Husband/partner has had drink (alcohol) problem, in last 2 years | Parent | no | substance household | 9yrs2m | 7 | 9 |
| pm1052 | Frequency father has taken cannabis/marihuana in last 2 years | Parent | no | substance household | 9yrs2m | 7 | 9 |
| pm1060 | Frequency father has taken amphetamines or other stimulants in last 2 years | Parent | no | substance household | 9yrs2m | 7 | 9 |
| pm1063 | Frequency father has taken heroin, methadone, crack, cocaine in last 2 years | Parent | no | substance household | 9yrs2m | 7 | 9 |
| pm3032 | Wife/partner has had drink (alcohol) problem, in last 2 years | Parent | no | substance household | 9yrs2m | 7 | 9 |
| n1057 | Mother has ever had a drug addiction | Parent | no | substance household | 8yrs1m | 8 | 8 |
| n1058 | Mother has ever had alcoholism | Parent | no | substance household | 8yrs1m | 8 | 8 |
| pl1057 | Respondent has ever had drug addiction | Parent | no | substance household | 8yrs1m | 8 | 8 |
| r2017 | Respondent has ever had a drug addiction | Parent | no | substance household | 11yrs2m | 10 | 11 |
| pq1022 | Partner has had an alcohol problem in the last two years | Parent | no | substance household | 12yrs1m | 10 | 12 |
| pq3032 | Partner's partner had a drink problem in the last 2 years | Parent | no | substance household | 12yrs1m | 10 | 12 |
| s1022 | Mother has had an alcohol problem in last 2 years | Parent | no | substance household | 12yrs1m | 10 | 12 |
| s3032 | Mother's partner has had an alcohol problem since study child's 10th birthday | Parent | no | substance household | 12yrs1m | 10 | 12 |
| pp2017 | Respondent has ever had a drug addiction | Parent | no | substance household | 11yrs2m | 11 | 11 |
| t5402 | Frequency respondent taken cannabis/marijuana in last two years | Parent | no | substance household | 2010 | 16.5 | 18.5 |
| t5406 | Frequency respondent taken cocaine in last two years | Parent | no | substance household | 2010 | 16.5 | 18.5 |
| t5410 | Frequency respondent taken amphetamines, ecstasy or other stimulants in last two years | Parent | no | substance household | 2010 | 16.5 | 18.5 |
| t5412 | Frequency respondent taken heroin, methadone, crack or other hard drug in last two years | Parent | no | substance household | 2010 | 16.5 | 18.5 |
| fjal4000 | Alcohol Use Disorders Identification Test (AUDIT) score | Child | no | substance household | 17.5yrs | 17.5 | 17.5 |
| t5510 | AUDIT total score | Parent | no | substance household | 2010 | 18.5 | 18.5 |
| fa5402 | Cannabis/marijuana | Parent | no | substance household | 2011-2013 | 18.5 | 20.5 |
| fa5406 | Cocaine | Parent | no | substance household | 2011-2013 | 18.5 | 20.5 |
| fa5410 | Amphetamines, ecstasy or other stimulants | Parent | no | substance household | 2011-2013 | 18.5 | 20.5 |
| fa5411 | Heroin, methadone, crack, other hard drug | Parent | no | substance household | 2011-2013 | 18.5 | 20.5 |
| fa5510 | AUDIT total score | Parent | no | substance household | 2011-2013 | 20.5 | 20.5 |
| ypa5005 | YPs age when partners have used physical force such as pushing, slapping, hitting or holding them down | Child | yes | violence child and partner | 0-21yrs | 0 | 18 |
| ypa5007 | YPs age when partners have used more severe physical force such as punching, strangling, beating them up, hitting them with an object | Child | yes | violence child and partner | 0-21yrs | 0 | 18 |
| ypa5009 | YPs age when YP's partners have pressured them into kissing/touching/something else | Child | yes | violence child and partner | 0-21yrs | 0 | 18 |
| ypa5011 | YPs age when partners have physically forced them into kissing/touching/something else | Child | yes | violence child and partner | 0-21yrs | 0 | 18 |
| ypa5013 | YPs age when partners have pressured them into having sexual intercourse | Child | yes | violence child and partner | 0-21yrs | 0 | 18 |
| ypa5015 | YPs age when partners have physically forced them into having sexual intercourse | Child | yes | violence child and partner | 0-21yrs | 0 | 18 |
| ypa5017 | YPs age when partners behaviour made them feel scared or frightened | Child | yes | violence child and partner | 0-21yrs | 0 | 18 |
| fg4422 | Someone they have been out with has intentionally slapped teenager | Child | no | violence child and partner | 13.5yrs | 13.5 | 13.5 |
| fg4424 | Someone they have been out with has intentionally kicked teenager | Child | no | violence child and partner | 13.5yrs | 13.5 | 13.5 |
| fg4428 | Someone they have been out with has intentionally pushed/grabbed/shoved teenager | Child | no | violence child and partner | 13.5yrs | 13.5 | 13.5 |
| fg4430 | Someone they have been out with has intentionally thrown something at teenager | Child | no | violence child and partner | 13.5yrs | 13.5 | 13.5 |
| fg4432 | Someone they have been out with has intentionally hit with their fist teenager | Child | no | violence child and partner | 13.5yrs | 13.5 | 13.5 |
| fg4435 | Another form of violence has been used against teenager in a romantic relationship | Child | no | violence child and partner | 13.5yrs | 13.5 | 13.5 |
| ypa5005_dup | YPs age when partners have used physical force such as pushing, slapping, hitting or holding them down | Child | no | violence child and partner | 0-21yrs | 18 | 21 |
| ypa5007_dup | YPs age when partners have used more severe physical force such as punching, strangling, beating them up, hitting them with an object | Child | no | violence child and partner | 0-21yrs | 18 | 21 |
| ypa5009_dup | YPs age when YP's partners have pressured them into kissing/touching/something else | Child | no | violence child and partner | 0-21yrs | 18 | 21 |
| ypa5011_dup | YPs age when partners have physically forced them into kissing/touching/something else | Child | no | violence child and partner | 0-21yrs | 18 | 21 |
| ypa5013_dup | YPs age when partners have pressured them into having sexual intercourse | Child | no | violence child and partner | 0-21yrs | 18 | 21 |
| ypa5015_dup | YPs age when partners have physically forced them into having sexual intercourse | Child | no | violence child and partner | 0-21yrs | 18 | 21 |
| ypa5017_dup | YPs age when partners behaviour made them feel scared or frightened | Child | no | violence child and partner | 0-21yrs | 18 | 21 |
| f242 | Physically hurt by PTNR >CH born | Parent | no | violence between parents | 8m | 0 | 0.67 |
| pd242 | Ptnr Physically Cruel Since Baby Born | Parent | no | violence between parents | 8m | 0 | 0.67 |
| ypa5050 | Amount YP has ever been aware of and affected by one 'parent' slapping, kicking, hitting or otherwise physically hurting the other | Child | yes | violence between parents | 21yrs | 0 | 21 |
| pc222 | PTNR physically hurt you since MID PREG | Parent | no | violence between parents | 8w | -1 | 0.16 |
| g322 | Partner physically cruel to mum >CH8MTHs | Parent | no | violence between parents | 1yrs9m | 0.67 | 2 |
| pe322 | Partner Physically Cruel | Parent | no | violence between parents | 1yrs9m | 0.67 | 2 |
| h232 | Whether partner was physically cruel to mum since study child was 18 months old and effect this had | Parent | no | violence between parents | 2yrs9m | 1.5 | 3 |
| pf5022 | Partner's partner was physically cruel to them since study child was 18 months old | Parent | no | violence between parents | 2yrs9m | 1.5 | 3 |
| j322 | PTR PHYS Cruel to MUM> CH 30 MTHs | Parent | no | violence between parents | 3yrs11m | 2.5 | 4 |
| pg3022 | Degree to which physical cruelty from a partner affected partner since child was 2.5 years old | Parent | no | violence between parents | 3yrs11m | 2.5 | 4 |
| k4022 | Mothers partner was physically cruel to her in past year | Parent | no | violence between parents | 5yrs1m | 4 | 5 |
| ph4022 | Respondent's assessment of how much their partner being physically cruel in the last year has affected them | Parent | no | violence between parents | 5yrs1m | 4 | 5 |
| l4022 | Respondent's partner was physically cruel to them since study child's 5th birthday | Parent | no | violence between parents | 6yrs1m | 5 | 6 |
| pj4022 | Respondent's assessment of how much partner being physically cruel since study child's 5th birthday has affected them | Parent | no | violence between parents | 6yrs1m | 5 | 6 |
| p2022 | Mother's husband/partner was physically cruel to her since the study child's 6th birthday | Parent | yes | violence between parents | 9yrs2m | 6 | 7 |
| pm2022 | Father's wife/partner was physically cruel to him since the study child's 6th birthday | Parent | yes | violence between parents | 9yrs2m | 6 | 7 |
| n3044 | Mother has ever kicked, bitten or hit partner with a fist | Parent | no | violence between parents | 8yrs1m | 8 | 8 |
| n3045 | Partner has ever kicked, bitten or hit mother with a fist | Parent | no | violence between parents | 8yrs1m | 8 | 8 |
| n3048 | Mother has ever physically twisted partner's arm | Parent | no | violence between parents | 8yrs1m | 8 | 8 |
| n3049 | Partner has ever physically twisted mother's arm | Parent | no | violence between parents | 8yrs1m | 8 | 8 |
| n3050 | Mother has ever tried to throw partner bodily | Parent | no | violence between parents | 8yrs1m | 8 | 8 |
| n3051 | Partner has ever tried to throw mother bodily | Parent | no | violence between parents | 8yrs1m | 8 | 8 |
| n3052 | Mother has ever beaten partner up | Parent | no | violence between parents | 8yrs1m | 8 | 8 |
| n3053 | Partner has ever beaten mother up | Parent | no | violence between parents | 8yrs1m | 8 | 8 |
| n3054 | Mother has ever tried to choke or strangle partner | Parent | no | violence between parents | 8yrs1m | 8 | 8 |
| n3055 | Partner has ever tried to choke or strangle mother | Parent | no | violence between parents | 8yrs1m | 8 | 8 |
| n3056 | Mother has ever threatened partner with a knife or other weapon | Parent | no | violence between parents | 8yrs1m | 8 | 8 |
| n3057 | Partner has ever threatened mother with a knife or other weapon | Parent | no | violence between parents | 8yrs1m | 8 | 8 |
| n3058 | Mother has ever used a knife or other weapon on partner | Parent | no | violence between parents | 8yrs1m | 8 | 8 |
| n3059 | Partner has ever used a knife or other weapon on mother | Parent | no | violence between parents | 8yrs1m | 8 | 8 |
| pl3044 | Respondent has ever bitten/kicked/hit their partner with a fist | Parent | no | violence between parents | 8yrs1m | 8 | 8 |
| pl3045 | Respondent's partner has ever bitten/kicked/hit them with a fist | Parent | no | violence between parents | 8yrs1m | 8 | 8 |
| pl3048 | Respondent has tried to twist their partner's arm | Parent | no | violence between parents | 8yrs1m | 8 | 8 |
| pl3049 | Respondent's partner has tried to twist their arm | Parent | no | violence between parents | 8yrs1m | 8 | 8 |
| pl3050 | Respondent has ever thrown/tried to throw their partner | Parent | no | violence between parents | 8yrs1m | 8 | 8 |
| pl3051 | Respondent's partner has ever thrown/tried to throw them | Parent | no | violence between parents | 8yrs1m | 8 | 8 |
| pl3052 | Respondent has ever beaten up their partner | Parent | no | violence between parents | 8yrs1m | 8 | 8 |
| pl3053 | Respondent's partner has ever beaten them up | Parent | no | violence between parents | 8yrs1m | 8 | 8 |
| pl3054 | Respondent has ever tried to choke their partner | Parent | no | violence between parents | 8yrs1m | 8 | 8 |
| pl3055 | Respondent's partner has ever tried to choke them | Parent | no | violence between parents | 8yrs1m | 8 | 8 |
| pl3056 | Respondent has ever threatened their partner with a knife/weapon | Parent | no | violence between parents | 8yrs1m | 8 | 8 |
| pl3057 | Respondent's partner has ever threatened them with a knife/weapon | Parent | no | violence between parents | 8yrs1m | 8 | 8 |
| pl3058 | Respondent has ever used a knife/weapon on their partner | Parent | no | violence between parents | 8yrs1m | 8 | 8 |
| pl3059 | Respondent's partner has ever used a knife/weapon on them | Parent | no | violence between parents | 8yrs1m | 8 | 8 |
| p2022_dup | Mother's husband/partner was physically cruel to her since the study child's 6th birthday | Parent | no | violence between parents | 9yrs2m | 8 | 9 |
| pm2022_dup | Father's wife/partner was physically cruel to him since the study child's 6th birthday | Parent | no | violence between parents | 9yrs2m | 8 | 9 |
| pp5022 | Respondent's wife/partner was physically cruel to them since the study child's 9th birthday | Parent | yes | violence between parents | 11yrs2m | 9 | 10 |
| r5022 | Respondent's husband/partner was physically cruel to them since study child's 9th birthday | Parent | yes | violence between parents | 11yrs2m | 9 | 10 |
| pp5022_dup | Respondent's wife/partner was physically cruel to them since the study child's 9th birthday | Parent | no | violence between parents | 11yrs2m | 11 | 11 |
| r5022_dup | Respondent's husband/partner was physically cruel to them since study child's 9th birthday | Parent | no | violence between parents | 11yrs2m | 11 | 11 |
| t3321 | Respondent's partner was physically cruel to respondent in last year | Parent | no | violence between parents | 2010 | 17.5 | 18.5 |
| fa3321 | In last year, partner was physically cruel to respondent | Parent | no | violence between parents | 2011-2013 | 20.5 | 20.5 |
