# Supplementary material for: Adverse childhood experiences in the children of the Avon Longitudinal Study of Parents and Children (ALSPAC)
Source: Wellcome Open Res. 2018 Aug 30;3:106. [Version 1] doi: 10.12688/wellcomeopenres.14716.1 (PMC6281007; doi:10.12688/wellcomeopenres.14716.1)
Supplement: Peer review history

# Peer review: Referee response for version 1

**DOI:** 10.21956/wellcomeopenres.16031.r33778

**Published:** 2018-12-06

**Authors:** Erin C Dunn

**Affiliations:** 1 Department Of Psychiatry, Harvard Medical School, Boston, MA, USA

Thank you for the opportunity to review this paper, which will be a terrific addition to the literature.  As already noted by the prior reviewers, this paper is written at the perfect level to guide future researchers in using the ALSPAC data to incorporate ACES into their work.  I especially commend the authors for their thoroughness, and transparency, which is reflected in every element of this paper. Comments below are minor in nature and aimed at helping to strengthen this already strong paper.

- In the last sentence of the Introduction, it would be helpful if the authors could add in additional language that helps to make clear the goals of a Data Note.  I make this comment because, having not been familiar until now with this type of paper, later statements in the paper read a bit awkwardly without this context.  I would therefore suggest a slight rephrasing to say something along the lines of:  “In this Data Note, we describe the processes used to derive the ACE measures in ALSPAC, and report the results of univariate and bivariate analyses to summarize these measures and their association with social-demographic factors.  We also provide practical guidance regarding multiple imputation strategies to employ when analyzing this ACE data as well as resources to better understand the ALSPAC data and how to access it, which we hope will be useful to both current and future users of ALSPAC data.
- In the Methods, please clarify this statement by perhaps adding “When the oldest children *in the cohort *were approximately 7 years of age”.  
- As information about triplets and quadruplet pregnancies is noted, it would also be helpful to report the number of twins in the sample.
- The “classic” ACES are defined in a way that makes clear the valence of the experience, whereas the “add-on” items are not worded as such.  I would consider possibly revising some of the labels where appropriate so that they are all worded negatively, consistent with the idea of these items tapping into adverse childhood experiences.
- The authors note: “The resulting 12083 questions were compared to previous articles using ALSPAC data to ensure that we had identified all relevant literature” – however, only 5 papers are then cited.  If the authors did search a more extensive set of literature, it might be helpful to future users to present a listing of those papers in the Supplemental Materials.  I also humbly suggest the authors consider citing one of my recent papers, which not only reported on a broad set of adversities, but also included a comprehensive strategy to address data missingness through multiple imputation ^1^.
- The authors use the terms “prospective” and “retrospective”, which although appear straightforward, are not so.  It would be helpful – especially for new users to this dataset – to indicate in a table footnote (such as in Table 1) how these concepts were defined.  Does prospective capture repeated events measured relatively close in time and within one year or two years of the initial event occurrence? Does retrospective capture cross-sectional assessments where participants are asked to report on the occurrence of an experience years or even a decade earlier? 
- Though I certainly could have missed it, it does not seem that the paper includes specific time points of assessment for each of the measures.  Given all of the detailed work that was put into making this accessible, these details would help even more to assist new users in working with these data.  The data that appears in the last table of the Supplemental Materials seems to possibly do that, though it is unclear what the authors mean by start time period and end tie period ( Supplemental Table 1)?  Although it would be tedious, it might be helpful to indicate more consistently the time frame that is captured through these measures in their description listed in Table 1.
- Under “Derive ACE measures” – I suggest a small wording change: “The R code used to derive these ACE measures in R will be supplied to *users at the time when data are requested from ALSPAC.  This code is also available, per request, for existing data users”*
- Is there a reason why EPDS scores are included both as socio-demographic factors and adversities, under “Multiple Imputation”?  
- It would be helpful if the authors could provide more detail in the supplement regarding the multiple imputation strategy.  The average reader will not be able to interpret a sentence like this: “Note the use of passive imputation for the ACE count scores and categorical ACE score variables, which are therefore constructed from the imputed ACE constructs and not used as an auxiliary variable for the imputation of the individual ACE constructs.”  If this Data Note is meant to serve as a guide for data users, it would be helpful to provide some basic introduction or literature for reference related to the type of imputation strategies employed (i.e., pointing out high-level differences between passive imputation, logistic regression, etc).  Otherwise, I worry these important details will be lost in translation. Relatedly, it would also be helpful to indicate how the imputed datasets were actually combined for analysis (or how point estimates were combined/averaged).
- Under “Patterns of Missing Data…”, please restate to “participants with more deprivation”. Please also note the two references in this section are unnumbered.
- In the “Dataset Validation” section – or in the supplement, please provide one sentence to clarify “subjective impact of ACE exposure”, as this would not unclear to most users. Relatedly, the authors note that there is a subset of children who have documented cases of maltreatment, but this data is not actually readily available (I have tried to access this data without success, as it sounds like the N’s are too small and there were some concerns about data confidentiality).  Consistent with the paper’s focus on transparency and practicality, it might be helpful to add in one additional sentence to provide a bit more context related to this issue and soften the hopefulness of that data being as rich as it now sounds.
- Under “Data Availability” section 3, would be helpful to add “ and what the costs are to access the data”, as data user costs vary widely across datasets. 
- In the Supplemental…
- What does Criterium dichotomization mean?  How it was asked?  Or whether it is possible to dichotomize the response?
- I would encourage the authors to add spaces in between the variables of the “Differences” tables (i.e., gender, social class, etc) or make the first column of this table wider to increase readability.

 

I have read this submission. I believe that I have an appropriate level of expertise to confirm that it is of an acceptable scientific standard.

## References

[1] DunnECSoareTWRaffeldMRBussoDSCrawfordKMDavisKAFisherVASlopenNSmithADACTiemeierHSusserES: What life course theoretical models best explain the relationship between exposure to childhood adversity and psychopathology symptoms: recency, accumulation, or sensitive periods?. *Psychol Med*.2018;48(15) : 10.1017/S0033291718000181 2562-2572 10.1017/S0033291718000181 29478418PMC6109629

# Peer review: Referee response for version 1

**DOI:** 10.21956/wellcomeopenres.16031.r34235

**Published:** 2018-12-03

**Authors:** Snehal M. Pinto Pereira

**Affiliations:** 1 UCL Research Department of Epidemiology & Public Health, London, UK

Thank you for asking me to review this paper. It is going to be extremely useful for future researchers planning on using measures of ACEs in ALSPAC. It is pitched at the right level, putting much of the detail in supplementary tables – so as not to distract from the main purpose (i.e. how ACE variables were generally measured in ALSPAC and a recommended strategy to deal with missing data). I appreciate the pragmatic approach to missing data taken because of issues re: lack of complete cases and high number of variables in the dataset. I commend the authors for their careful consideration to multiple imputation and the appropriate guidance given for future researchers using this data. I do have some, mostly minor, queries that I detail below.

  

1. I would urge the authors to consider referencing papers or government documents on the conventional definitions of some of the ACEs considered in the manuscript and how the measures used from ALSPAC compare to these definitions. See, for example Gilbert et al Lancet. 2009 ^1^ and Her Majesty's Government; Department for Education. Working together to safeguard children. 2015 ^2^. This information can be added as an extra column in supplementary table 2. Considering these standard definitions, I query whether “Partner/respondent was physically cruel to child” is specific enough to be considered physical abuse. I also wonder if the authors would consider whether “child never felt loved”, currently part of the parent-child bond, would be better placed as a component of neglect (which is defined in the 2015 document to include neglect of, or unresponsiveness to, a child’s basic emotional needs).
2. A footnote could be added to Table 2 explaining how mean (SE)/% is calculated for the imputed data e.g. where these calculated for each imputed dataset and then averaged across the imputed datasets?
3. I recommend a quick re-read of the supplementary section to ensure that all the table numbers etc are accurate (e.g. the section starts with supplementary table 2, and then in the missing data section references supplementary table 1, but presents supplementary table 3).
4. How were the binary ACE measures actually created? E.g. for sexual abuse, 6 items were considered (assuming no missing data) – how were these items combined together to create the binary ‘sexual abuse’ variable.
5. Supplementary Figure 1 is, in my view, important and a sub-section of it could be considered as a figure in the main text. Both researchers and practitioners are interested in which ACEs are correlated and which aren’t (and, for example, it is interesting to note that the high correlation between physical abuse and emotional abuse is observed in other datasets/generations).
6. In a few places, clarity of text can be improved e.g. “Xix: violence between child and partner”, could be re-phrased to be “violence between the child and their partner”.

I have read this submission. I believe that I have an appropriate level of expertise to confirm that it is of an acceptable scientific standard.

## References

[1] GilbertRWidomCSBrowneKFergussonDWebbEJansonS: Burden and consequences of child maltreatment in high-income countries. *Lancet*.2009;373(9657) : 10.1016/S0140-6736(08)61706-7 68-81 10.1016/S0140-6736(08)61706-7 19056114

[2] Her Majesty's GovernmentDepartment for Education: Working together to safeguard children. *Appendix A*.2015; Reference source

# Peer review: Referee response for version 1

**DOI:** 10.21956/wellcomeopenres.16031.r33780

**Published:** 2018-09-27

**Authors:** Helen Minnis

**Affiliations:** 1 Institute of Health and Wellbeing, University of Glasgow, Glasgow, UK

This is a very clear and “researcher-friendly” paper which will be extremely useful for future users of ALSPAC data.  It describes a careful search within ALSPAC for variables that offer useful information for constructing both “classic” Adverse Childhood Experiences variables as well as several additional ones.  This has allowed construction of a “classic” ACE scale as well as an “extended” ACE scale and categorical versions of both are also offered.  Detailed information has been supplied about the way multiple imputation was used to derive more realistic ACE prevalences, since the proportion of the ALSPAC cohort with enough ACE data to construct actual ACE scores differs socio-demographically from the entire cohort.  The supplementary material is useful and, in general, the right things are kept for supplementary files so that the paper itself flows nicely.

 Specific points are below – all are minor and are mainly points of clarity.

 Page 3: Please clarify “violence between child and partner”? Reader might think “mother’s partner”.  Would be helpful to say “violence between child and child’s partner”? Becomes clearer in “definitions” but useful to mention here.

 Was parental death too rare to include? Might be worth mentioning. Also I notice there isn’t a “physical neglect” question as in the original Felitti questionnaire. Not necessarily a problem but worth mentioning early on. 

 Page 4:  When participants, in their 20s, retrospectively reported on “violent behaviour of their own partner”, do you know during what age range?  Presumably under 18 but do you know what the lowest age was?  I guess – if this occurred largely, say, between age 14 and 18, one could argue this was not really “childhood”.

 Page 5, second column, para 2 where you talk about categories of the extended ACE score, can you say a bit more here to justify why you picked those particular categories?

 This section is confusing and needs more clarification: “However, note that questions that span a larger time window (e.g. exposure 0–11 years or  0–16 years) than the period of interest (e.g. 0–8 years) would be excluded from ACE construct calculations.”

 The section on multiple imputation, especially the illustration on page 6 of imputation, is likely to be very helpful for future ALSPAC-users.

 Page 6, second column first para: might be worth expanding this paragraph a bit by commenting more on some of results in Figure 1 supplementary material.  You might want to mention both the highest correlations and also some very apparently uncorrelated variables such as emotional neglect.  I think a lot of practitioners and clinicians will want to know what is correlated with what and may be surprised at some of this.

 I wonder if the title of your Dataset Validation section might be expanded a bit to “Dataset validation, strengths and limitations” because you do seem to expand into general limitations here.

 Tiny wording points:

 Abstract: “the high dimensional” – should this be “the highly dimensional”?

 Thank you for asking me to review this very helpful paper.

I have read this submission. I believe that I have an appropriate level of expertise to confirm that it is of an acceptable scientific standard.
